# DNA-directed termination of RNA polymerase II transcription

**DOI:** 10.1016/j.molcel.2023.08.007

**Published:** 2023-09-07

**Authors:** Zhong Han, George A. Moore, Richard Mitter, David Lopez Martinez, Li Wan, A. Barbara Dirac Svejstrup, David S. Rueda, Jesper Q. Svejstrup

**Affiliations:** 1 Department of Cellular and Molecular Medicine, Panum Institute, University of Copenhagen, Blegdamsvej 3B, 2200 Copenhagen N, Denmark; 2 Mechanisms of Transcription Laboratory, The Francis Crick Institute, 1 Midland Road, London NW1 1AT, UK; 3 Single Molecule Imaging group, MRC-London Institute of Medical Sciences, and Section of Virology, Department of Infectious Disease, Faculty of Medicine, Imperial College London, London W12 0NN, UK; 4 Bioinformatics and Biostatistics, The Francis Crick Institute, 1 Midland Road, London NW1 1AT, UK

## Abstract

RNA polymerase II (RNAPII) transcription involves initiation from a promoter, transcriptional elongation through the gene, and termination in the terminator region. In bacteria, terminators often contain specific DNA elements provoking polymerase dissociation, but RNAPII transcription termination is thought to be driven entirely by protein co-factors. We used biochemical reconstitution, single-molecule studies, and genome-wide analysis in yeast to study RNAPII termination. Transcription into natural terminators by pure RNAPII results in spontaneous termination at specific sequences containing T-tracts. Single-molecule analysis indicates that termination involves pausing without backtracking. The “torpedo” Rat1-Rai1 exonuclease (XRN2 in humans) greatly stimulates spontaneous termination but is ineffectual on other paused RNAPIIs. By contrast, elongation factor Spt4-Spt5 (DSIF) suppresses termination. Genome-wide analysis further indicates that termination occurs by transcript cleavage at the poly(A) site exposing a new 5′ RNA-end that allows Rat1-Rai1 loading, which then catches up with destabilized RNAPII at specific termination sites to end transcription.

## Graphical abstract

**Graphical abstract:**
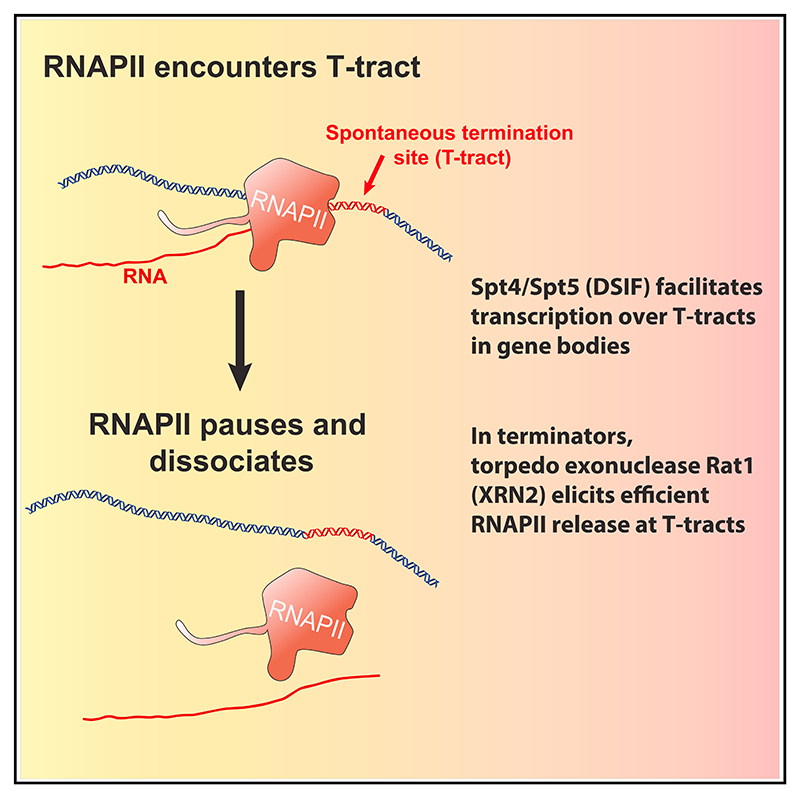

## Introduction

Transcription initiation at promoters and termination at terminators define the boundaries of a protein-coding gene. Although the importance of precise transcriptional initiation is obvious, correct transcriptional termination is also essential for correct regulation of gene expression, for example, by preventing interference between transcription units.^1–3^ Likewise, although the biochemical and structural basis of RNA polymerase II (RNAPII) transcription initiation is well established,^4–6^ the precise mechanism of termination remains unclear. This is at least partly because RNAPII termination, in contrast to that of all other RNA polymerases, effectively occurs in two phases. First, termination of the mRNA transcript (“transcript termination”) occurs in a sequence-dependent manner through site-specific RNA cleavage and addition of a polyadenylation (poly(A)) tail (the mRNA transcript cleavage site is thus also called the poly(A) site). Importantly, because the mRNA is cleaved and polyadenylated, its 3′-end does not correspond to the site of RNAPII termination. Instead, the polymerase continues transcribing but then terminates in the downstream terminator region (“transcription termination”). Although separate from transcript termination, efficient transcription termination requires co-transcriptional mRNA transcript cleavage.^1,2^

Because of the high stability of the ternary elongation complex (TEC) comprising RNA polymerase, DNA template, and RNA transcript,^7–9^ dismantling it in terminators may be as challenging as assembling it at promoters. In bacteria, which do not have transcript cleavage and poly(A) tailing as an intrinsic feature, two general mechanisms for transcription termination have been defined: intrinsic termination and Rho-dependent termination.^3^ Intrinsic termination is mediated by signals encoded by the DNA template, whereas Rho-dependent termination relies on the RNA translocase Rho, which binds the nascent RNA and dissociates the TEC. As indicated above, termination of RNAPII transcription in eukaryotes is more complex and less well understood. Two different mechanisms have been proposed. The first is the so-called torpedo model, which posits that the new, unprotected 5′ RNA-end resulting from transcript cleavage at the poly(A) site provides an entry point for the exonuclease Rat1 (XRN2 in humans), which degrades the nascent RNA, catches up with the polymerase, and dissociates it.^10–15^ The second is the allosteric change model, which proposes that transcription past the poly(A) site causes the dissociation of anti-termination factors and/or a conformational change within RNAPII, which somehow promotes termination.^16–19^ Unified working models have also been proposed.^13,20–24^ Somewhat disconcertingly, however, the efficiency of Rat1-dependent transcription termination with purified RNAPII *in vitro* is either very low^14^ or Rat1 does not induce the polymerase to terminate at all.^25^ Likewise, the precise molecular basis for allostery in transcriptional termination remains unclear.

Due to the lack of robust and well-defined *in vitro* transcription termination systems, these models are mostly based on observations *in vivo*, and a role for DNA sequence has only been reported in now longstanding mapping of individual sites of RNAPII termination, which uncovered a poorly defined role for T- and sometimes A-tracts in the human c*-myc* and *beta-globin* genes.^26–28^ Indeed, exactly how natural terminator sequences affect transcription by RNAPII has not been investigated. In the hope of settling “the endless quarrels at the end of genes,”^29^ we used *in vitro* RNAPII elongation assays with pure yeast proteins to investigate whether and how the highly stable RNAPII TEC can be disassembled. Our results show that spontaneous but highly Rat1-Rai1 responsive termination of RNAPII transcription occurs at certain T-rich (non-template/coding strand) sequences in natural terminators of protein-coding genes. Further evidence for the existence of site-specific termination was obtained by genome-wide analysis in budding yeast. Together, our data provide compelling support for a general model for transcription termination by RNAPII.

## Results

### Spontaneous RNAPII termination

**Figure 1:**
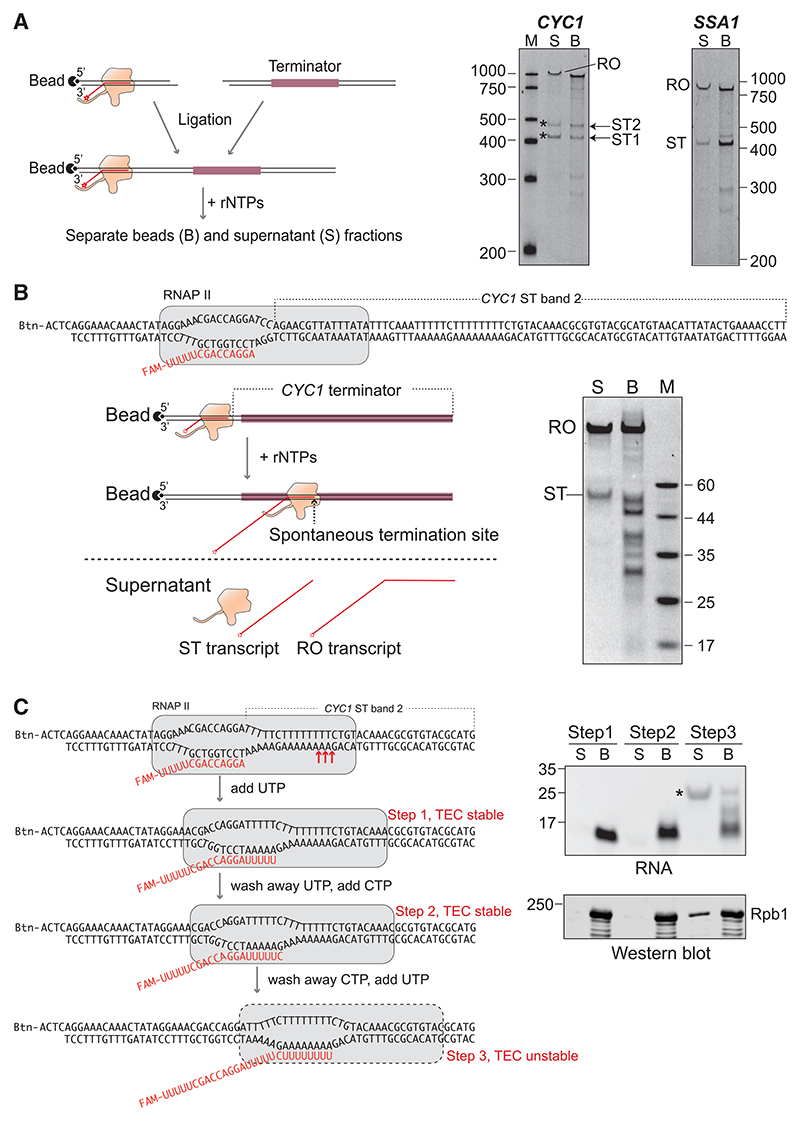
RNAPII spontaneous termination *in vitro* (A) Left, experimental scheme. Not to scale, terminator fragments are 0.8–1 kb long. Right, 5′-end FAM-labeled transcripts from supernatant (S) and bead fraction (B) visualized after denaturing 6% TBE-urea PAGE. RO, runoff; ST, spontaneous termination; M, size marker. Asterisks indicate spontaneous termination bands. (B) Upper left, short, oligonucleotide-based transcription template, containing *CYC1* spontaneous termination band 2. Btn, biotin; red text, 5′ FAM-labeled RNA; lower left, experimental scheme. TECs dissociated at ST sites are released into the supernatant and separated from the beads; lower right, As in (A), but resolved by 10% denaturing TBE-urea PAGE. (C) Left, experimental scheme, step walking of RNAPII on *CYC1* spontaneous termination transcription template, as in (B). Red arrow indicates sites of ST. Right, upper, 5′ FAM-labeled RNA transcripts from RNAPII step walking experiment visualized as in (B). Lower, Rpb1 subunit of RNAPII from step walking experiment revealed by western blot. See also Figure S1.

To investigate the effect of terminator sequences on RNAPII transcript elongation, we used an *in vitro* system, with transcript elongation starting directly from a pre-assembled TEC containing only highly purified *Saccharomyces cerevisiae* (*S. cerevisiae*) RNAPII (Figure S1A), DNA, and RNA. Such TECs have previously been shown to faithfully emulate normal transcript elongation, including the integrity of the transcription bubble during both forward and retrograde movement.^9,30^ With this simple system, we transcribed into *S. cerevisiae CYC1* terminator regions of varying size and with increasing resolution (Figure 1, schematics on left; see also Figure S1D). Transcription from the bead-immobilized, pre-assembled RNAPII TEC was initiated by the addition of NTPs, after which the reaction was split into a bead fraction containing TECs, and a supernatant fraction containing free transcripts and RNAPII released due to TEC dissociation. Transcription through a template containing the *CYC1* terminator primarily gave rise to “full-length” transcripts where the polymerase had reached the end of the ~1 kb DNA template (Figures 1A and S1D), with some TECs still remaining intact near the end and others having RNAPII runoff, releasing the transcript into the supernatant.

More importantly, evidence of transcription pausing was also observed, especially in the area of the DNA template encompassing the *CYC1* terminator sequence (Figure 1A, beads, indicated by arrows). Intriguingly, a substantial fraction of TECs appeared to dissociate at these sites and release the transcript to the supernatant fraction (Figure 1A, supernatant, indicated by asterisks). Similar results were observed with the *SSA1* terminator (Figure 1A; see also other terminators below). The TEC is normally extremely stable^8,9,30^; therefore, its unforced dissociation during transcript elongation through a terminator in the absence of termination factors was surprising.

A time course of the reaction showed that the spontaneous termination (ST) sites were located near sites of prolonged RNAPII transcription pause/arrest sites (Figure S1B; see also Figure S1F). At pausing sites, RNAPII is prone to backtracking and arrest, but elongation factor TFIIS promotes RNAPII-mediated transcript cleavage, which allows resumption of transcription.^31^ Indeed, the response to TFIIS addition has previously been used to show that what was first thought to be termination was actually reversible pausing.^32^ We therefore tested the effect of TFIIS. Although addition of TFIIS caused the disappearance of the RNA products characteristic of pausing and backtracking (Figure S1C, lane 2 vs. lane 4), the level of spontaneous RNAPII termination was not affected (Figure S1C, lane 1 vs. lane 3).

By comparing ST sites with RNA markers (Figure 1A), we estimated the position of the pausing and termination sites in the *CYC1* terminator. One of these sites, ST1, is upstream of the *CYC1* transcript cleavage site *in vivo* and was therefore not investigated further. To map ST2 more precisely, TECs were assembled with DNA oligonucleotides based on the region around it, on which dissociation of the TEC again occurred at a specific site (Figures 1B, S1D, and S1E). We mapped the termination site to the end of a long T-tract in the non-template/coding strand, whereas transcriptional pausing happened throughout that T-tract (Figure S1F). Intriguingly, intrinsic termination signals in bacteria are characterized by a series of U residues in the transcribed RNA and a GC-rich stem-loop structure upstream of this poly-U region.^33^ There is no such hairpin structure upstream of the poly-U tracts in the *CYC1* ST site, and ST occurs even when transcription is initiated immediately upstream of the terminator sequence (Figures S1E and S1F; see also Figure 1C), showing that TEC dissociation at this site does not require a secondary structure in the nascent RNA. This also holds true for other spontaneous RNAPII termination sites tested below.

To characterize ST more precisely, we performed stepwise “walking” of the TEC across the termination site, with addition of only the relevant next nucleotide and separation of the reaction products at each step (Figure 1C). As expected, RNAPII TECs are normally extremely stable, with neither transcript nor RNAPII being released from the TEC in the first two steps of RNAPII progression. By contrast, in step 3, when RNAPII runs across the long T-tract, a substantial fraction of TECs dissociate, resulting in the nascent RNA and RNAPII being released into the supernatant (Figure 1C), indicating that the TEC, indeed, becomes unstable specifically at the termination site. In agreement with the idea that RNAPII TECs are unstable over this specific DNA sequence, we have been unable to assemble a TEC *de novo* with an RNA oligonucleotide specifically targeting RNAPII to this site.

### Nucleic acid requirements for efficient spontaneous termination

**Figure 2:**
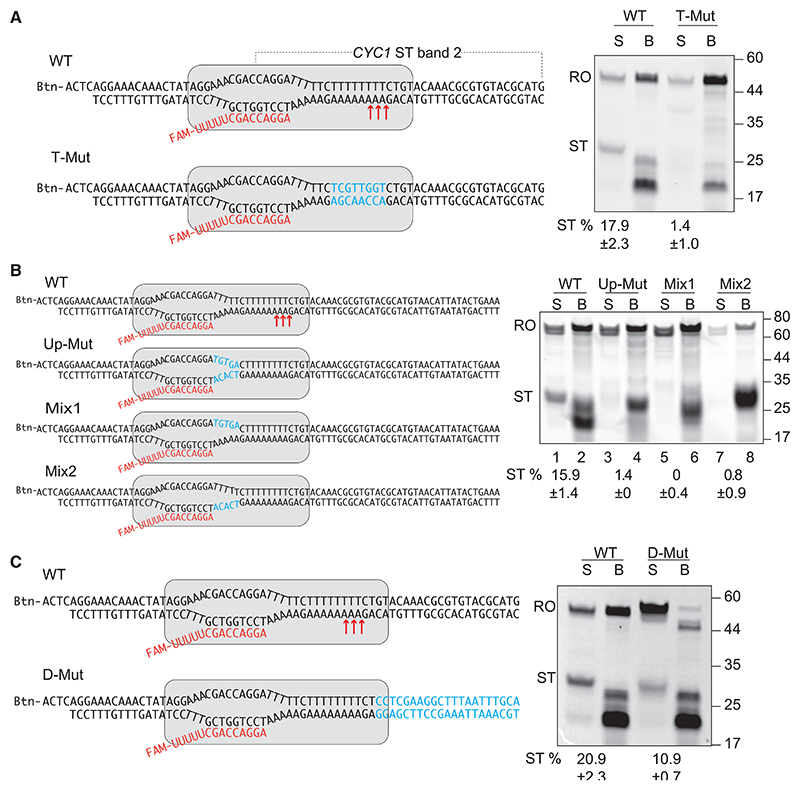
Sequence requirement for ST Left, schemes and template sequences. Red arrows indicate the sites of ST. Altered sequences in light blue. Right, 5′-end FAM-labeled RNA transcripts from *in vitro* transcription assay visualized as in Figure 1. ST efficiency calculated as ratio of ST band in supernatant fraction versus the full-length bands. See also Figures S1G and S2.

Although an analysis of the CYC1 terminator might not be generally transferable to all terminators, mutation analysis was nevertheless performed to determine the main features of this specific terminator sequence. It seemed obvious that the T-tract in the non-template strand might be critical for RNAPII termination. Indeed, disrupting it significantly diminished termination (Figures 2A and S1G). Gradually reducing its length also gradually decreased the efficiency of RNAPII dissociation so that little termination was observed with a 5-nucleotide T-tract (Figure S2A). Because RNAPII interacts with DNA upstream and downstream of the transcription bubble,^7,34,35^ we asked whether the DNA sequences flanking the T-tract are important. Interestingly, mutation of the upstream TTTTT to TGTGA largely eliminated ST (Figure 2B, lane 1 vs. lane 3). This effect could be due to either DNA sequence or an effect of the nascent transcript. To try and distinguish between these possibilities, we substituted only the non-template strand TTTTTT sequence to TGTGA, while preserving the original template strand (Figure 2B, Mix1). Little or no termination was observed for this sub-strate, suggesting that UUUUU in the nascent RNA upstream of the termination site is not in itself adequate to trigger dissociation. A non-template sequence of TTTTT (tested by changing only the template sequence from AAAAA to ACACT) is also not in itself enough to allow release of the TEC at the termination site, but this change did give rise to high levels of pausing in the area around the site (Figure 2B, Mix2).

Somewhat surprisingly, we found that changing the DNA sequence downstream of the long poly-T-tract reduced ST efficiency as well (Figure 2C). This might be due to the downstream sequence somehow affecting TEC stability. Indeed, step walking of RNAPII showed that in step 3, where RNAPII pauses at the ST site, the paused TEC was relatively more stable when the downstream sequence is mutated (Figure S2B).

In summary, these data indicate that, although the long T-tract is indispensable for ST, the flanking sequences are also important for destabilizing the TEC. Similar results were observed at a ST sequence from the *TAH1* termination region (Figure S2C). Moreover, ST occurred more efficiently at low NTP (Figure S2D). Given that transcription rates are decreased under such conditions,^36^ this suggests that low elongation rates favor ST. Mammalian RNAPII purified from calf thymus also spontaneously terminated transcription at yeast terminator sequences (Figure S2E), indicating that RNAPII’s ability to spontaneously terminate is conserved.

Consecutive polyA-T base pairs (A-tracts) cause DNA bending,^37^ and the DNA sequence A4T4 shows DNA bending similar to 8 As, whereas T4A4 does not.^38^ If DNA bending alone were responsible for ST, it would thus be expected that an A4T4 mutant would show normal termination, whereas the T4A4 mutant would not. However, the A4T4 mutant showed reduced ST, and even less than the T4A4 mutant (Figure S3A). Placing the A-tract on the non-template strand also decreased termination (Figure S3B). Because inversion of A/T-tracts does not affect DNA bending,^37^ these results indicate that bending of the A/T-tract is not a main cause for termination. More work is required to fully understand the general sequence- and structural features triggering ST by RNAPII.

### Single-molecule FRET assay to visualize spontaneous termination

**Figure 3:**
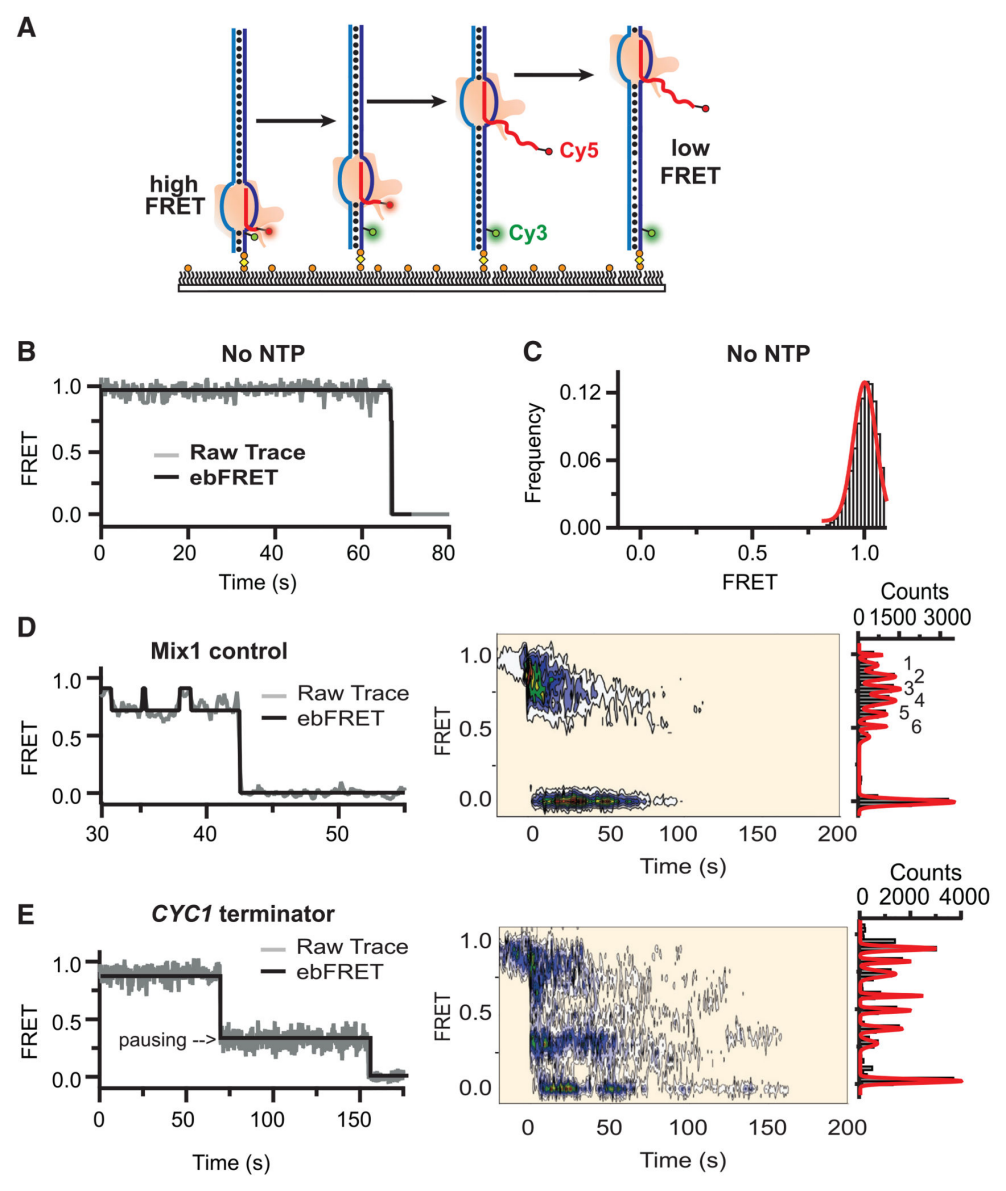
Single-molecule FRET analysis of termination (A) Schematic of smFRET assay. (B) Example of FRET trace in the absence of transcription (no NTPs). (C) FRET histogram in the absence of NTPs. Histogram fitted to Gaussian (red fit). (D) Data for the control sequence (“Mix1”). Left, FRET time trace example of transcribing RNAPII on the control template. ebFRET fitted data shown in black. Middle, post-synchronization histogram of actively transcribing molecules. See Figure S4 for examples where polymerases did not finish transcription (final FRET ≠ 0). Right, histogram of idealized FRET determined from HMM analysis showing the range from high FRET (0.9) to 0.0 FRET. Individual peaks fitted to a Gaussian distribution (red fit) and numbered. (E) Data for the *CYC1* termination sequence, presented as in (D). Notice the different x axes in (D) and (E), left. See also Figure S4.

To gain further insight into the mechanism of sequence-dependent ST, we developed a single-molecule fluorescence resonance energy transfer (FRET) assay (smFRET) to visualize transcription dynamics on a DNA molecule containing the ST site, or, as a control, a mutated sequence that shows significant pausing but little dissociation of the TEC (*Mix1* sequence from Figure 2B). Surface immobilized TECs were assembled with a fluorescently labeled FRET donor (Cy3) on the template strand and a FRET acceptor (Cy5) at the 5′-end of the RNA primer (Figures 3A and S4A). In the absence of NTPs, the FRET pair is in proximity, yielding static trajectories with high (~0.95) FRET values (Figures 3B and 3C). In the presence of NTP, the transcribing RNAPII relocates the growing RNA chain away from the slide surface, thus increasing the distance of the FRET pair, resulting in a FRET decrease. The transition begins when RNAPII starts elongation stochastically after the addition of NTPs, and then elongation either decreases the FRET signal to zero (complete transcription) or to a paused intermediate state, which may remain stable, backtrack to higher FRET, or eventually continue to complete transcription (FRET signal zero). On the Mix1 control sequence (N_tot_ = 108), 35% of the trajectories exhibited quick (<50 s) stepwise decreases from high to 0 FRET upon addition of NTP, indicating complete transcription (example in Figure 3D). 35% remained static (FRET ~0.95; no transcription), 20% moved forward but then “backtracked” between ~0.6 and ~0.95 FRET, and the remaining 10% were stably stalled at 0.2–0.4 FRET (see Figure S4B for example).

In the subsequent analysis, we focused on the trajectories that exhibited complete transcription. Hidden Markov model analysis of these trajectories shows that on the Mix1 control sequence, the polymerase transitions through several intermediate states in a mostly unidirectional and stepwise manner with occasional backtracks, as expected for transcribing TECs. Indeed, a histogram of the observed FRET values shows that RNAPII may transition through at least six intermediate states between 0.8 and ~0.5 FRET (Figure 3D, right).

On the *CYC1* termination substrate(N_tot_ = 109; fraction of transcribing polymerases 24%), it takes longer to complete transcription (>50–100 s) (Figure 3E). More importantly, these trajectories exhibit a prominent pause at ~0.3 FRET not observed with the control sequence, followed by a drop to zero FRET (Figure 3E, middle). Importantly, no evidence of backtracking in these traces was observed. These observations are consistent with the TEC disassembling prematurely due to ST. Using labeled RNA primers of increasing length, we determined empirically that the 0.3 FRET state corresponds to ~13^th^ or 14^th^ nucleotide from the starting site, consistent with the termination site observed in the bulk biochemical experiments. Hidden Markov model analysis confirms that approximately half of these trajectories exhibit long (50–60 s) pauses around ~0.3–0.4 FRET (Figure 3E, right).

Together, these data indicate that RNAPII pauses 6–7 nucleotides into the long T-tract, stalls, and then falls off the DNA. This is consistent with a model in which transcription resulting in ST involves a long period of pausing, but with little or no backtracking immediately prior to TEC dissociation.

### Elongation factor Spt4/5 suppresses spontaneous termination

**Figure 4:**
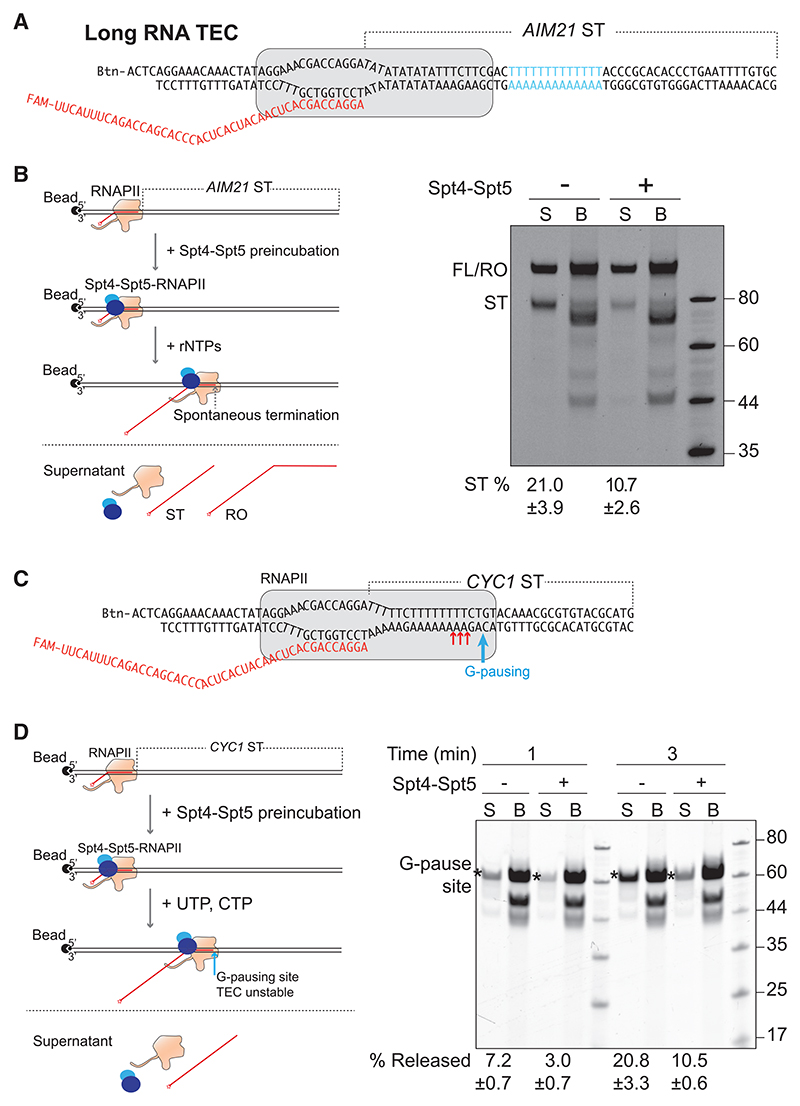
Elongation factor Spt4-Spt5 complex suppresses termination (A) Sequences used for TEC assembly. Blue, T-tract. (B) Left, experimental scheme. Right, analysis of termination using 5′-end FAM-labeled RNA, visualized by 10% TBE-urea polyacrylamide gels. FL/RO, full length/runoff. (C) Sequences and scheme for (D). Red arrow indicates sites of ST. Blue arrow indicates G-pausing site. (D) Left, TEC was assembled and preincubated with Spt4-Spt5, followed by UTP and CTP addition to initiate elongation, for RNAPII to either terminate (supernatant), or pause at the G-pausing site (on beads). Right, 10% TBE-urea PAGE analysis of transcripts. Note that relatively more RNAPII remains bound to the template at the G-site in the presence of Spt4/5, indicating that it stabilizes the elongation complex to read past the site. See also Figure S5.

It seems likely that there will be mechanisms to repress unwarranted ST during transcript elongation through T-tracts inside genes. We showed above that elongation factor TFIIS is unable to prevent ST (Figure S1C) but also tested the effect of purified Spt4-Spt5 (DSIF in humans; Figure S5), which increases RNAPII processivity.^39^ It has previously been reported that stable interaction between Spt4-Spt5 and the RNAPII TEC requires a long nascent RNA.^40^ For these and other reasons, a ST site from the *AIM21* terminator was initially used (Figures 4A and 4B, left). When Spt4-Spt5 complex was added to TECs, it markedly decreased ST (Figure 4B, right). Little or no effect on ST was observed when using short RNA transcripts (Figure S5), consistent with previous reports.^40^ Similar experiments with the *CYC1* termination site indicated that Spt4-Spt5 may suppress ST by stabilizing the paused elongation complex near the termination site (Figures 4C and 4D).

### Cooperation between RNAPII ST and Rat1-dependent termination

**Figure 5:**
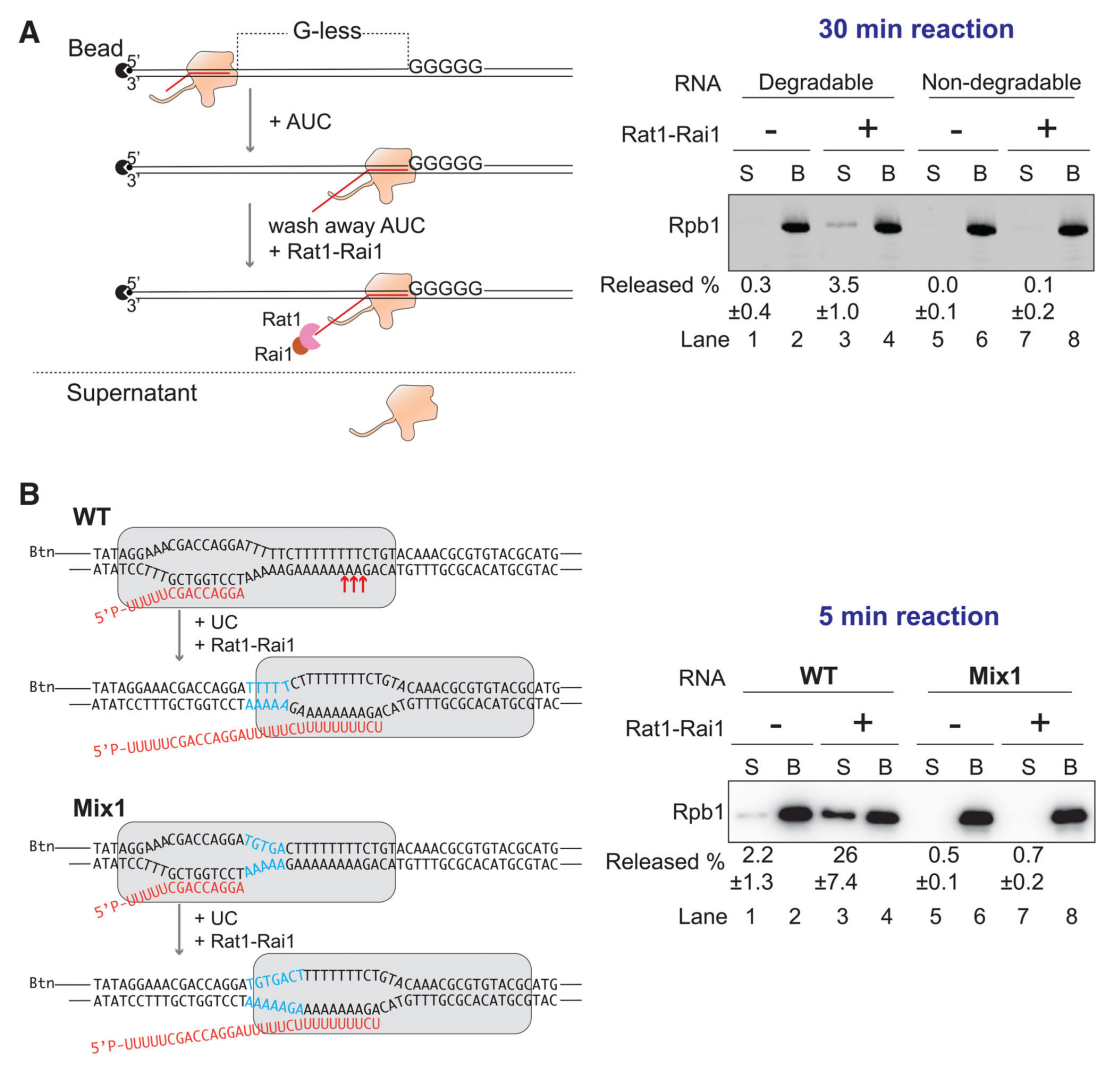
Cooperation between spontaneous and Rat1-dependent termination (A) Left, experimental scheme. Right, after incubation with Rat1-Rai1, reactions were analyzed by Rpb1 western blotting. See Figure S6B for RNA composition. (B) Left, sequences and experimental scheme. Red arrows, sites of ST. Right, termination analyzed by Rpb1 western blotting. See also Figure S6.

The present model for transcription termination posits that after co-transcriptional transcript cleavage by the cleavage and polyadenylation factor (CPF in yeast; CPSF in humans), RNA exonuclease Rat1 (Xrn2 in humans) degrades the nascent transcript from the 5′-end to catch up with and dissociate the RNAPII TEC (the torpedo model^10,11,15^). However, Rat1-dependent transcription termination with purified RNAPII *in vitro* is either very inefficient^14^ or the polymerase is not induced to terminate at all.^25^ We hypothesized that termination sites might be needed to cooperate with Rat1 and its associated regulator Rai1 to elicit efficient termination. Because Rat1-Rai1 degrades the nascent RNA, termination and TEC dissociation was in these experiments detected by Rpb1 western blot. Using purified Rat1-Rai1 complex (Figure S6A), we first confirmed that termination efficiency is, indeed, very low with stopped RNAPII TECs on substrates that do not harbor a termination site (Figure 5A, lane 3), consistent with previous data.^14,25^ Moreover, by using a non-degradable chimeric RNA (Figure S6B), we also found that blocking 5′-3′ RNA degradation abolishes Rat1-dependent transcription termination, providing support for the torpedo model: the Rat1 exonuclease, indeed, requires 5′-3′ degradable RNA to elicit termination (Figure 5A, lane 7 vs. lane 3; see also Figure S6B). To test how Rat1-Rai1 affects termination efficiency at the *CYC1* terminator sequence, we again compared it with the Mix1 control sequence that shows little ST. For these experiments, we first determined conditions that induce only minimal levels of ST in the absence of Rat1-Rai1, or on the control sequence in the presence of Rat1-Rai1. Under these conditions, the termination sequence alone (Figure 5B, lane 1), or addition of Rat1-Rai1 (Figure 5B, lane 7) result only in minimal dissociation of RNAPII TECs. However, when testing termination at the ST sequence in the presence of Rat1-Rai1, the combination triggered high levels of dissociation of RNAPII (Figure 5B, lane 3). Importantly, given that Mix1 sequences show elevated levels of transcriptional pausing without giving rise to termination (Figure 2B), RNAPII pausing is clearly insufficient for torpedo-mediated termination *in vitro* (Figure 5B, lane 7). Together, these results point to a model in which the torpedo exonuclease catches up with an already destabilized form of RNAPII at sites that have a propensity to trigger ST. The combined action of these changes in the RNAPII TEC then triggers highly efficient, Rat1-stimulated termination of transcription.

Together, the results above point to a model in which accessory factors such as Rat1-Rai1 and Spt4-Spt5 either stimulate or inhibit termination at specific sites harboring paused, de-stabilized RNAPII TECs.

### T-tract termination sequences play an important role *in vivo*

The results obtained by reconstitution biochemistry above suggest that termination sites containing T-tracts may be key components of the termination mechanism. If so, such T-rich sequence motifs should mainly locate to termination regions and, indeed, long T-tracts are enriched downstream of open reading frame (ORFs) (Figure S7A). We also analyzed the sequence composition around yeast *in vivo* termination areas previously defined by Cramer and co-workers^41^ and found that T-tracts are enriched at such sites (Figures S7B and S7C).

**Figure 6:**
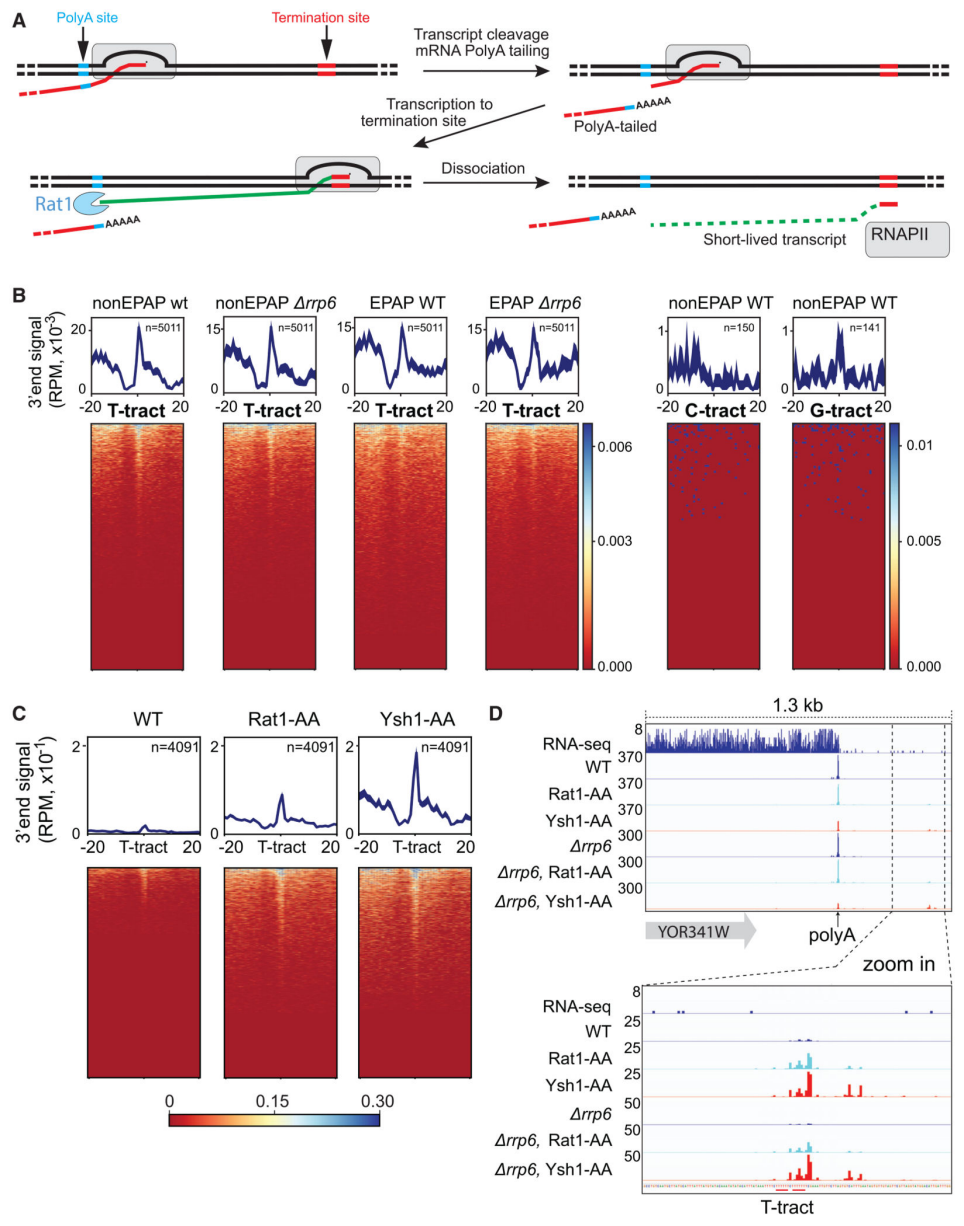
RNAPII transcription termination is facilitated by T-tracts *in vivo* (A) Schematic of RNAPII behavior at termination. Nascent RNA (green) generated upon transcript cleavage is highly unstable (co-transcriptionally degraded by Rat1). (B) Heatmap and metagene profile of 3′-end sequencing signals aligned to T-tracts (≥6 mer) (left), G-tracts (≥6 mer) or C-tracts (≥6 mer) (right) in termination regions (canonical poly(A) +1 kb downstream). 3′-end sequencing performed in WT strains and *Δrrp6* strains, with or without *in vitro* polyadenylation by *E. coli* poly(A) polymerase (EPAP), as indicated. Experiments (n = 3) were merged. (C) Experiments in WT, Rat1-AA, and Ysh1-AA strains. Heatmap and metagene profile of 3′-end sequencing signals aligned to 3′-end of T-tracts (≥6 mer) in termination regions. Experiments (n = 2) were merged. (D) Single-gene examples. Data from a previously published RNA-seq dataset^46^ are shown at the top to frame the position of the canonical poly(A) sites. Other lanes represent 3′-end sequencing results. See also Figures S7−S11.

To more directly investigate exactly where RNAPII transcription termination occurs *in vivo*, we performed next-generation sequencing of 3′-ends (3′-end sequencing) in different *S. cerevisiae* yeast strains. In our approach, a short period of incubation of cells in 4-thiouracil (4TU) was followed by isolation of poly(A)-tailed RNA species, both with and without prior poly(A) tailing *in vitro*. This procedure was used in an attempt to enrich for the short, terminated transcripts produced by RNAPII after passing the poly(A) site (Figure 6A), based on approaches previously described by others.^42–44^ Enrichment of nascent transcripts was necessary to avoid merely sequencing the ends of the stable and abundant mRNAs and was attempted in several different yeast strains. For example, an *rrp6* deletion was used to inactivate the nuclear exosome that degrades non-coding RNA.^45^ The results obtained were generally of high quality with good reproducibility between samples (Figure S8A).

In support of the idea that termination occurs at T-tracts, 3′ end transcript signals did pile up when aligned to T-tracts but not to G- or C-tracts (Figure 6B). Somewhat surprisingly, similar results for RNAPII termination at nucleotide tracts at the end of protein-coding genes were found in wild type (WT) and *Δrrp6* and with and without *in vitro* poly(A) by *E. coli* poly(A) polymerase (EPAP) prior to library production (Figure 6B), suggesting that these transcripts already contained a poly(A) tail. By contrast, differences at cryptic unstable transcripts (CUTs) and sn/snoRNA genes were detected (Figures S8B–S8E). Importantly, the presence of poly(A) tails in the nascent transcripts from protein-coding genes confirms that they arose because of termination rather than, for example, RNAPII pausing. It is also worth noting that little 3′ end signal was observed when aligning to T-tracts in ORFs (Figure S9A), consistent with the idea that unwanted termination at T-tracts encountered during normal transcript elongation through gene bodies is suppressed.

Rat1 exonuclease or transcript cleavage factor Ysh1 (CPSF73/CPSF3 in humans) was now removed by anchor away^47^ in the presumption that this would either stabilize the short transcripts downstream of the transcript cleavage site (Rat1 depletion) or generate longer, more stable ones through the reduction in transcript cleavage at the poly(A) site (Ysh1 depletion)^41^ (Figure S9B). The results obtained from this approach were generally of high quality with good reproducibility between samples (Figure S9C). As expected, anchoring away Ysh1 from the nucleus resulted in a decrease of the 3′ end signal mapping to the canonical cleavage and poly(A) sites of protein-coding genes (Figures S9D and S9E, upper). More importantly, the 3′ end signal at T-tracts increased markedly (Figures 6C, 6D, and S9E, lower), indicating that transcripts released at T-tracts were, indeed, due to termination rather than transcript cleavage by Ysh1 but also suggesting that spontaneous transcription termination sites may serve as a general mechanism for RNAPII termination *in vivo*. We note that no correlation between the expression level of genes and their order in the heatmap was uncovered. Likewise, no clear features were found for these genes. Similarly, we detected no compelling genomewide relationship between termination efficiency and the length of the T-tracts.

Upon Ysh1-mediated RNA cleavage, Rat1 degrades nascent RNA, catches up with RNAPII and induces transcription termination. Therefore, we might also expect an increase of 3′ end signals at T-tract when impeding Rat1 function. Indeed, we found more 3′ end signal at T-tracts when anchoring away Rat1 as well (Figures 6C, 6D, and S9E, lower), indicating that RNAPII also terminates at T-tracts without Rat1. Importantly, although termination at T-tracts is much more efficient in the presence of transcript cleavage and the Rat1 torpedo, the transcripts produced by RNAPII downstream of the poly(A) site are rapidly co-transcriptionally degraded by Rat1 and therefore extremely difficult to detect in WT cells (see Figure 6A). The apparent increase in termination at T-tracts upon Ysh1- or Rat1 Anchor away thus does not mean that termination at T-tracts predominantly (or only) occurs in the absence of Ysh1 or Rat1 but merely reflects the much longer half-life of nascent RNA-produced downstream of poly(A) sites under those conditions so that such termination events can be detected (green in Figure 6A). Indeed, pulse-chase experiments showed that transcripts released at T-tracts are relatively stable in Ysh1-anchor-away cells (Figure S9F). Single-gene examples verified the enrichment of 3′ end signals at T-tracts after Rat1- or Ysh1 anchor away (Figures 6D and S10), and similar results for Ysh1- and Rat1 anchor away were obtained in the *Δrrp6* background (Figure S11A).

Interestingly, although not relevant to the main focus of this study, we found that Rat1 appears to be generally important for degrading introns (Figure S11B), consistent with previous data on single-gene examples.^48–50^

**Figure 7:**
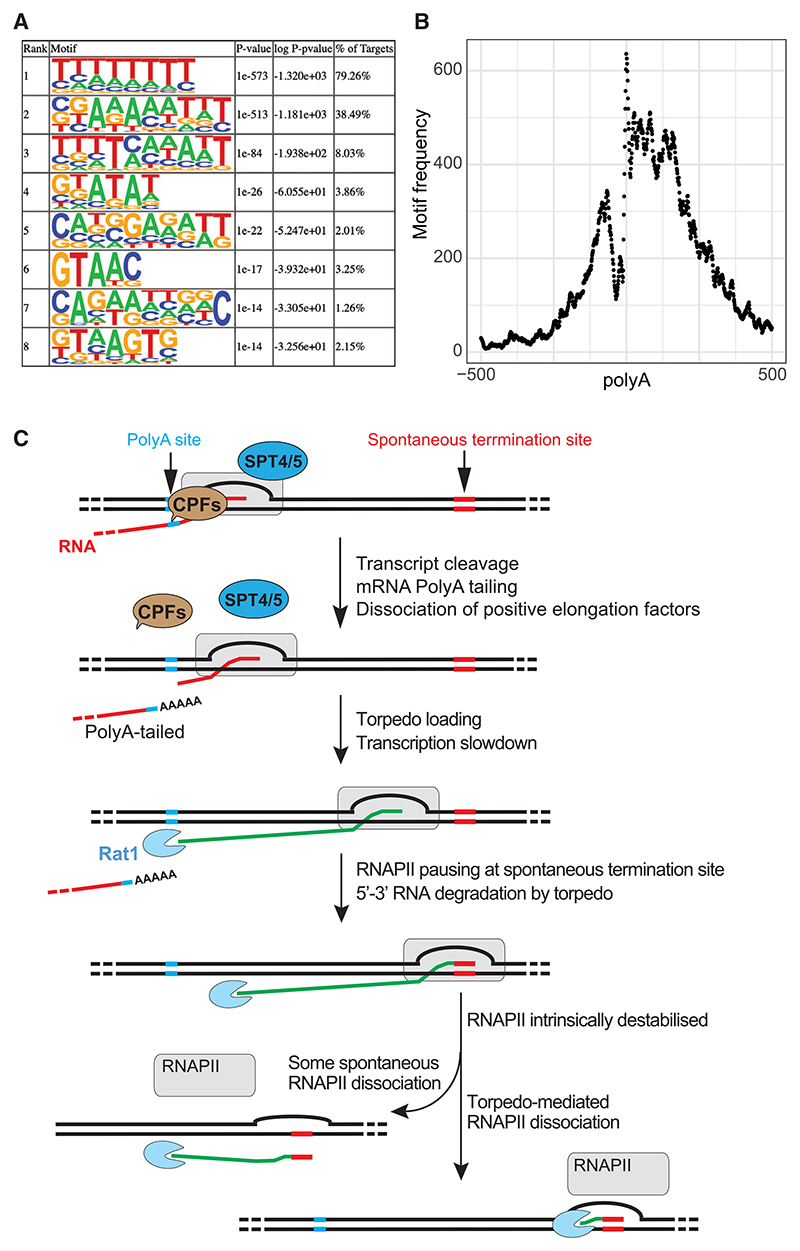
The mechanism of RNAPII transcription termination (A) Motif discovery results produced by *de novo* motif discovery using 3′-end sequencing data. Because a given sequence can be matched to multiple motifs, motif hits found are not mutually exclusive and percentages do not add up to 100%. (B) Location of the consensus motif1 from (A) surrounding the poly(A) sites. (C) Step-by-step model for transcriptional termination by RNAPII. CPFs, cleavage and polyadenylation factors.

In the analysis of the genome-wide data above, the alignment to T-tracts was justified by the results of our reconstitution experiments with terminators *in vitro*. However, to investigate in an unbiased way at which sequence motifs ST occurs most frequently *in vivo*, we now used *de novo* motif discovery in the 3′-end sequencing data. Here, we calculated the ratio of the coverage in the Ysh1AA and WT samples (Ysh1AA/WT). We then isolated islands of read-depth coverage where this ratio is >2 and that simultaneously resides in the 1 kb of sequence downstream of protein-coding genes (n = 6,600). This yielded 13,466 candidate target regions, which were used for motif analysis.^51^ Remarkably, such analysis showed that by far the most significant sequence motif at transcription termination sites enriched in Ysh1 cells is a T-tract (Figure 7A). Indeed, more than 79% of target regions (10,673 of 13,466 regions) contain the T-rich 8mer motif shown at the top. We note that it is possible for a 1 kb downstream region of a gene to contain multiple such hits. If we instead took a “gene-centric” view, we found that 2,870 genes (~44% of all genes) display 1 or more such “T-tract termination” events, providing further strong support for the idea that RNAPII termination frequently occurs at such sites *in vivo*. As expected, the position of termination motifs in the terminator of a gene is not fixed; sites are widely distributed over the first 250 bp downstream of the poly(A) site (Figure 7B).

## Discussion

Current models for RNAPII termination are almost entirely focused on the Rat1-Rai1 (XRN2) torpedo and other protein co-factors that regulate transcript elongation; no significant role for the DNA sequence of terminators is envisioned. Using a mixture of biochemistry, single-molecule experiments, and genome-wide analysis in yeast, we here provide evidence that the eukaryotic RNAPII elongation complex dissociates at specific sites in natural terminators containing T-tracts. Our data thus support a general model for transcription termination by RNAPII in which the stability of the elongation complex is intrinsically altered in terminators by the sequence of the DNA template being transcribed, empowering the Rat1-Rai1 torpedo (Figure 7C). Our results also provide a compelling explanation for the enrichment of T-tracts in transcriptional terminators and their scarcity in the coding regions of genes.

### The mechanism of spontaneous termination

It has long been known that RNA polymerase pausing directed by template sequence is an important component of intrinsic termination in bacteria.^3^ Briefly, such pausing is triggered by the formation of an unstable RNA:DNA hybrid upon synthesis of an RNA U-tract, which, in turn, provides time for an upstream terminator hairpin to form, which then invades the RNAP RNA exit channel to trigger TEC destabilization and dissociation.^3^ Interestingly, however, sequence-directed termination at T-tracts has also been described for archaeal RNAP and eukaryotic RNA polymerase III (RNAPIII).^21,52–55^

Importantly, besides the hairpin termination mechanism proposed for bacteria, the RNA:DNA hybrid may also in itself play a general, critical role in maintaining RNAP TEC stability.^9^ In this connection, it is worth noting that an rU/dA pairing is several magnitudes less stable than rA/dT,^56^ which might explain why we only observe spontaneous termination at T-tracts. Our data show that, unlike in bacteria, ST by RNAPII does not require an upstream RNA hairpin, whereas sequences flanking the T-tract of the *CYC1* terminator do affect termination efficiency. We speculate that some sites of ST may have evolved to be particularly efficient through optimization of such flanking sequences. Importantly, rather than requiring a specific DNA sequence or motif, we believe ST is more likely to be driven by DNA structure. It is thus easy to imagine how a particular DNA structure capable of destabilizing the RNAPII elongation complex might be under-pinned by a range of different DNA sequences, and likely most efficiently by those containing long T-tracts. The precise characteristics of such destabilizing DNA configurations await structural analysis.

Intriguingly, our single-molecule experiments show the existence of a unique RNAPII pausing state at the termination site, which is abolished upon replacing sequences upstream of the T-tract. The flanking sequences might thus affect ST efficiency by affecting the stability and/or the pausing characteristic of the TEC at T-tracts. Importantly, the single-molecule experiments further showed that little or no RNAPII backtracking is detected prior to ST, consistent with our other experiments showing that TFIIS has no effect on termination efficiency. This suggests that transcriptional backtracking and ST are uncoupled events: some polymerases stop and backtrack in the T-tract and can be reactivated by TFIIS, whereas a different group pauses and then terminates. We note that intrinsic bacterial terminator sequences will also function optimally only in the absence of backtracking because any retrograde motion by RNAP would disrupt hairpin formation.

### Termination by RNAPII, a brief history

After reconstitution of RNAPII transcription initiation in cell-free systems had been achieved in the early 80s, considerable effort was invested in understanding the mechanism of the process *in vitro*.^57^ In studies on transcript elongation, prior to the discovery of TFIIS and the critical role played by transcript cleavage at poly(A) sites in transcriptional termination, data consistent with “intrinsic termination” by RNAPII *in vitro* were reported.^58–60^ However, the subsequent discovery of TFIIS and its ability to often rescue “termination” at such sites^32,61^ meant that the idea that RNAPII might spontaneously disengage at certain DNA sequences was largely abandoned, or at least generally forgotten. More recently, others have shown that RNAPII cannot spontaneously dissociate at certain bacteriophage λ- or RNAPIII intrinsic terminators containing T-tracts when there is no hairpin upstream of the T-tract.^55,62^ Likewise, although RNAPIII is known to spontaneously dissociate at certain T-rich sequences, structural work on RNAPIII pre-termination complexes suggested that sequence-dependent termination may be unique to this polymerase, i.e., not possible with RNAPI or RNAPII.^63,64^

The current perception that transcription termination by RNAPII is governed only by protein co-factors^1,2,65^ is not due only to the confusion about the evidence for sequence-directed termination but arguably more to the unique way in which transcript termination at the poly(A) site was found to be connected to transcriptional termination by RNAPII. Indeed, alongside the early *in vitro* studies of transcription, ground-breaking *in vivo* studies showed how RNA processing is tightly coupled to the process of transcription: both mRNA splicing and cleavage of transcripts at the poly(A) site are co-transcriptional, with several CPFs associated with the RNAPII-specific C-terminal domain (CTD) in a phosphorylation-specific manner.^66^ The realization that cleavage at the poly(A) site is required for transcription termination and especially the later discovery of the role played by Rat1/XRN2 RNA exonuclease in RNAPII termination^10,11^ thus effectively muted the discussion of sequence-directed termination, although an undefined role for the terminator region has been reported in mapping of individual sites of RNAPII termination, some of which uncovered T-tracts in the human c*-myc, beta-globin*, and a few snRNA genes but without providing a mechanism.^26–28,67^

### RNA degradation by Rat1

Our experiments with WT, Rat1-, and Ysh1-anchor-away cells nicely illustrate the co-transcriptional and transcript cleavage-dependent degradation of transcripts performed by Rat1 in yeast. Indeed, although a clear indication of termination at T-tracts could be detected also in WT cells, it was much stronger in cells where the nucleus was depleted for Rat1 or transcript cleavage factor Ysh1. We do not believe this means that T-rich sequences merely function as a “fail-safe” mechanism for RNAPII termination; instead, we suggest that termination happens at such DNA sequences also in WT cells. The data on Rat1 function and termination at T-tracts *in vitro* strongly support this idea. Unfortunately, given that Rat1-mediated degradation of the nascent transcript occurs to the extent that torpedoing of RNAPII comprises an integral part of the termination mechanism, the RNA evidence required to support this contention *in vivo* is in effect destroyed by the termination machinery. Interestingly, the transcripts terminated at T-tracts in Ysh1-anchoraway cells were remarkably stable (Figure S9E). Whether this is due to the co-transcriptional poly(A) tailing of such spontaneously terminated transcripts in the absence of transcript cleavage, for example, by the poly(A) polymerase or the TRAMP complex,^68^ is unclear.

We also note that Rat1 anchor away had a significant effect on the degradation of practically all introns (*S. cerevisiae* only has ~300 such introns) (Figure S11B). Given that Rat1 function is not abrogated in the anchor-away technique but merely depleted from the nucleus by conditional tethering to an abundant cytoplasmic protein (the “anchor” being a subunit of the large ribosome subunit^47^), our data also provide evidence for significant, general debranching and rapid Rat1-dependent degradation of excised introns in the nucleus of yeast cells.

### A cohesive, general model for transcription termination by RNAPII incorporating terminator DNA elements

With the data reported here, it is possible to propose a cohesive model for RNAPII transcriptional termination, which explains the importance of all the known fundamental forces impinging on the terminating polymerase, namely, the rate of elongation (which can be affected by CTD phosphorylation and association with co-factors), the Rat1/XRN2 torpedo, and allosteric effects on the RNAPII TEC (Figure 7C). Indeed, the findings reported here provide direct evidence for intrinsic allosteric effects on the polymerase itself playing an important role in RNAPII transcription termination.

Rat1/XRN2 is critical for termination *in vivo*.^10,11,20,22,69^ Like-wise, dynamic dephosphorylation of RNAPII and transcription factors takes place upon RNAPII encountering poly(A) signals, which, in turn, results in decreased RNAPII transcription rates.^13,23,24,70,71^ Such decreased rates were proposed to render RNAPII a “sitting duck” for Rat1 to displace from the transcription template.^13^ However, the efficiency of Rat1-induced RNAPII transcription termination is surprisingly low *in vitro*.^14,25^ Indeed, our data corroborate the idea that stopping or slowing down RNAPII in itself does little to enable Rat1-mediated dissociation of the TEC. Rather, we found that extensive RNAPII termination is triggered only when combining Rat1 with termination sites, supporting an integrated model in which RNAPII slows down upon passing the poly(A) site, which not only allows Rat1 torpedo to catch up^12,72,73^ but also increases the likelihood of complying with termination sites, at which Rat1 becomes hyper-functional. In further support of this model, we found that slow elongation, brought about by decreasing NTP levels, itself enhances ST *in vitro* as well. Conversely, given that even coding regions sometimes contain T-tracts, one might expect there to be a mechanism to prevent unwanted termination during normal transcript elongation. In support of this idea, general elongation factor Spt4-Spt5 impedes ST, at least partly by stabilizing the TEC at T-tracts, providing an additional potent mechanism for regulating sequence-directed termination of transcription. Other positive elongation factors may further enhance this effect.

### Limitations of the study

This study uses a mixture of *in vitro* reconstitution and *in vivo* approaches in yeast to provide evidence that termination of RNAPII transcription often occurs at DNA sequences containing T-tracts. Although clear evidence for termination at such tracts was obtained *in vitro* and in WT cells *in vivo*, it was much more evident in yeast cells depleted for the transcript cleavage factor Ysh1 (CPSF73/CPSF3 in humans) or the torpedo exonuclease Rat1 (XRN2). Rather than indicating that RNAPII dissociation at specific termination sequences merely functions as a “fail-safe” mechanism in the absence of these protein factors, we believe that termination may typically occur at such DNA sequences, also in WT cells. However, direct evidence for this contention is difficult to obtain. Indeed, termination involves mRNA transcript cleavage and (co-transcriptional) Rat1-mediated degradation of the nascent RNA, which by its very nature removes the RNA evidence for the precise site of RNAPII dissociation from the template: the process of termination itself removes the RNA evidence for it.

It is important to note that we do not contemplate that a specific, conserved sequence motif is required for DNA-encoded termination. It is more likely that certain DNA sequences adopt a structure that can stop and destabilize the RNAPII elongation complex so that it can be dissociated. In this model, such structures are readily adopted by sequences containing T-tracts. Mutational analysis and/or DNA structure determination will be required to support this idea. It is also worth emphasizing that although such structures must themselves greatly destabilize RNAPII because they have a great effect in reconstituted transcription reactions *in vitro*, they might additionally affect other DNA-associated events inside cells, including the binding and behavior of DNA-binding proteins, such as nucleosomes. More work will be required to investigate these possibilities.

## Star⋆Methods

Detailed methods are provided in the online version of this paper and include the following:

- KEY RESOURCES TABLE
- RESOURCE AVAILABILITY
  - Lead contact
  - Materials availability
  - Data and code availability
- EXPERIMENTAL MODEL AND STUDY PARTICIPANT DETAILS
  - Bacteria strains
  - Yeast strains and culture conditions
  - Insect cells
- METHOD DETAILS
  - Plasmid construction
  - Yeast RNAPII purification
  - Rat1-Rai1 purification
  - Spt4-Spt5 expression and purification
  - Purification of long DNA oligonucleotides for use in transcription *in vitro*
  - Production of fluorescently labelled RNA marker
  - *In vitro* transcription and termination
  - Western blot
  - Denaturing PAGE Purification of DNA and RNA Oligonucleotides for smFRET
  - Slide passivation and smFRET
  - Single-Molecule Trajectories Analysis – ebFRET, TDP and PSH
  - RNA 3’ end sequencing
- QUANTIFICATION AND STATISTICAL ANALYSIS
  - Poly-A/C/T/G tract sequence analyses of the *Saccharomyces cerevisiae* genome
  - Poly-A/C/T/G tract sequence analyses of termination sites
  - 3’ end sequencing alignment, quantification and normalization
  - Related annotation files
  - Heatmaps and metagene profiles
  - Motif analysis of spontaneous termination sites

## Star⋆Methods

### Key Resources Table

**Table:** 

REAGENT or RESOURCE	SOURCE	IDENTIFIER
Antibodies		
Anti-RNAPII subunit Rpb1 (8WG16)	Cell services, The Francis Crick Institute	N/A
Donkey Anti-mouse, HRP conjugate	Jackson ImmunoResearch	Cat # 715-035-151; RRID: AB_2340771
Donkey Anti-mouse, Alexa Fluor 680 conjugate	Invitrogen	Cat # A10038; RRID: AB_2534014
Bacterial and virus strains		
NEB 5-alpha Competent E. coli One	NEB	Cat # C2988J
BL21-CodonPlus (DE3)-RIPL	Thermo Fisher Scientific	Cat # NC9122855
DH10Bac Competent Cells	Thermo Fisher Scientific	Cat # 10361012
Chemicals, peptides, and recombinant proteins		
complete EDTA-free protease inhibitor cocktail tablets	Sigma-Aldrich	Cat # 11873580001
3xFLAG peptide	Peptide Chemistry,The Francis Crick Institute	N/A
ANTI FLAG M2 agarose	Sigma-Aldrich	Cat # A2220
Mono Q™ 5/50 GL	Cytiva	Cat # 17516601
Vivaspin 20 centrifugal concentrator	Sigma-Aldrich	Cat #Z614661
NheI-HF	NEB	Cat #R3131L
NotI-HF	NEB	Cat #R3189L
NcoI-HF	NEB	Cat #R3193L
BamHI-HF	NEB	Cat #R3136L
HindIII-HF	NEB	Cat #R3104L
BbsI-HF	NEB	Cat # R3539L
PolyA Polymerase from E.coli	NEB	Cat # M0276L
fast dna ladder	NEB	Cat # N3238S
Q5 Hot Start High-Fidelity DNA polymerase	NEB	Cat # M0494L
PrimeSTAR HS with GC buffer	Takara	Cat # R044B
HisTrap FF Crude	Cytiva	Cat # 11000458
HiTrap Heparin HP	Cytiva	Cat # 17040601
Superose 6 Increase 10/300 GL	Cytiva	Cat #29091596
Fast Flow Q Sepharose column	Cytiva	Cat # 17-0510-01
MicroSpin G-25 Columns	Cytiva	Cat # 27532501
Grace’s Insect Medium	Thermo Fisher Scientific	Cat # 11595030
Expifectamine Sf transfection reagent	Thermo Fisher Scientific	Cat #A38915
Sf-900 III insect media	Thermo Fisher Scientific	Cat # 12658027
Dynabeads Myone Streptavidin T1	Thermo Fisher Scientific	Cat # 65602
RNase T1	Thermo Fisher Scientific	Cat # EN0541
Invitrogen 0.1 to 2kb RNA Ladder	Thermo Fisher Scientific	Cat # 11518766
RNA marker templates	Thermo Fisher Scientific	Cat # AM7782
NuPAGE™ 4 to 12%, Bis-Tris	Thermo Fisher Scientific	Cat #WG1403A
Amicon Ultra-4 Centrifugal Filter Unit	Merckmillipore	Cat #UFC8100
PVDF Membrane	Merckmillipore	Cat # IPFL00010
4-thiouracil	Sigma-Aldrich	Cat # 440736
Monoclonal ANTI-FLAG M2 antibody	Sigma-Aldrich	Cat # F3165-1MG
Rapamycin	Sigma-Aldrich	Cat # R0395
MTSEA biotin-XX linker	Biotium	Cat # BT90066
Maxtract high density (200x2ml)	Qiagen	Cat # 129056
Micro biospin P-30 gel columns	Bio-rad	Cat # 732-6250
RNase OUT	Invitrogen	Cat # 10777-019
Intercept (PBS) Blocking Buffer	LI-COR	Cat # 927-70001
Fluorescein-12-UTP	Jena bioscience	Cat # NU-821-FAMX
Critical commercial assays		
Pierce™ 660nm Protein Assay Reagent	Thermo Fisher Scientific	Cat # 22660
RNeasy minElute kit	QIAGEN	Cat # 74204
μMACS Streptavidin Kit	Miltenyi Biotec	Cat # 130-074-101
Invitrogen™ RiboMinus™	Thermo Fisher Scientific	Cat # 10388792
Transcriptome Isolation Kit, yeast		
QuantSeq 3’ mRNA-Seq Library Prep Kit (REV)	Lexogen (Nordic Biolabs)	Cat # 16.24
MAXIscript T7 transcription kit	Thermo Fisher Scientific	Cat #AM1312
Deposited data		
RNA 3’end sequencing	GEO Datasets	GEO: GSE218928
Experimental models: Cell lines		
Sf9	Oxford Expression Technologies	Cat # 600100-SF9 cells
Experimental models: Organisms/strains		
Rpb3-flag::NAT, prb1Δ	Kind gift from John Diffley	N/A
HHY168, MATalpha tor1-1	Euroscarf	Cat # Y40343
fpr1::NAT RPL13A-2xFKB12::TRP1		
Rat1 FRB, HHY168 RAT1-FRB::KAN	Baejen et al.^41^	N/A
Ysh1 FRB, HHY168 YSH1-FRB::KAN	Baejen et al.^41^	N/A
rrp6Δ::His5 (s.pombe) in HHY168	this study	N/A
rrp6Δ::His5 (s.pombe) in Rat1 FRB	this study	N/A
rrp6Δ::His5 (s.pombe) in Ysh1 FRB	this study	N/A
Oligonucleotides		
See Table S1 for Oligonucleotides		N/A
Recombinant DNA		
pET21b Rat1-His	This study	pZH81
pET28a-Rai1	This study	pZH82
Dual G-less cassette	Genscript	pZH1
Dual G-less containing yCYC1 polyA	This study	pZH20
Dual G-less containing ySSA1 polyA	This study	pZH23
pLIB	Weissmann et al.^74^	RRID: Addgene_80610
pLIB-8His3C-spt4	This study	pZH61
pLIB-spt5	This study	pZH60
pYES2	Thermo Fisher Scientific	Cat # V82520
Software and algorithms		
ImageQuant	Cytiva	https://www.cytivalifesciences.com/
Cutadapt v2.10	Kechin et al.^75^	https://cutadapt.readthedocs.io/en/stable/
STAR v2.7.6a	Dobin et al.^76^	https://github.com/alexdobin/STAR/
Samtools v1.122	Danecek et al.^77^	http://www.htslib.org/
deepTools v3.3.1	Ramirez et al.^78^	https://deeptools.readthedocs.io/en/develop/
BEDTools V2.27.1	Quinlan^79^	https://bedtools.readthedocs.io/en/latest/
Kent Tools2	Kent et al.^80^	N/A
HOMER suite v4.10.42	Heinz et al.^51^	http://homer.ucsd.edu/homer/motif/
Galaxy EU server	Galaxy	usegalaxy.eu
ebFRET	van de Meent et al.^81^	https://github.com/ebfret
SMFRET analysis	Brenlla et al.^82^	https://github.com/singlemoleculegroup
Adobe Photoshop	Adobe	https://www.adobe.com/au/products/photoshop.html
Adobe Illustrator	Adobe	https://www.adobe.com/uk/products/illustrator.html

## Resource Availability

### Lead contact

Further information and requests for resources and reagents should be directed to and will be fulfilled by the lead contact, Dr. Jesper Q. Svejstrup (jsvejstrup@sund.ku.dk).

### Materials availability

This study did not generate unique reagents, but any wanted material can be obtained by contacting the lead contact.

## Experimental Model And Study Participant Details

### Bacteria strains

E.coli strains BL21-CodonPlus (DE3)-RIPL were grown in standard LB media at 37°C supplemented with appropriate antibiotics (Ampicillin (100 μg/ml)-Chloramphenicol (34 μg/ml)).

### Yeast strains and culture conditions

All *Saccharomyces cerevisiae* strains used in this study are derivatives of W303 and were grown at 30°C in YPD media (1% yeast extract, 2% bactopeptone, and 2% glucose) and manipulated using standard techniques^83^. Where indicated, rapamycin (final concentration at 0.1 mg/ml) was added to anchor away Rat1 or Ysh1 for one hour. Genotypes of all yeast strains are provided in the key resources table.

### Insect cells

Insect cells (Sf9) were maintained at 27 °C in Sf900 III medium (GIBCO).

## Method Details

### Plasmid construction

pZH81: Rat1 was PCR amplified using oligos oZH435 and oZH436 from yeast genomic DNA and ligated into pET21b (Addgene, 69741-3) backbone (double digestion with Nhe1 and Not1), containing a C-terminal His tag. pZH82: Rai1 was PCR amplified using oZH437 and oZH438 from yeast genomic DNA and ligated into pET28a (Addgene, 69864-3) backbone (double digestion with NcoI and NotI). pZH61: Spt4 was PCR amplified using oZH390 and oZH392 from yeast genomic DNA and ligated into pLIB backbone^84^ (double digestion with BamHI and HindIII). pZH60: Spt5 was PCR amplified using oZH388 and oZH389 from a Spt5 expressing plasmid^40^ (gift from Joseph Reese) and ligated into pLIB (Addgene, 80610) backbone (double digestion with BamHI and HindIII). pZH20: CYC1 terminator sequence was amplified using oZH111 and oZH112 from pYES2 plasmid (ThermoFisher Scientific, V82520) and ligated into dual G-less cassette (Genscript synthesized) plasmid backbone (pZH1 digestion with BamHI and NotI). pZH23: SSA1 terminator sequence was amplified using oZH127 and oZH128 from yeast genomic DNA and ligated into the same dual G-less cassette plasmid backbone.

## Yeast RNAPII purification

100L of yeast *S. cerevisiae* expressing Rpb3-flag (in Δ*prb1* strain) was cultured in YPD media and harvested at OD_600_ 5-10. The pellet was resuspended in 1/10 pellet volume with lysis buffer (50 mM HEPES pH 7.6, 400 mM ammonium sulfate, 10 mM MgSO_4_,1 mM EDTA, 10% glycerol, Protease inhibitor Roche cocktail tablet) and broken by freezer mill. The resulting powder can be stored at -80°C for several years. For purification (at 4°C unless otherwise noted), an appropriate amount of powder was resuspended in a similar volume of lysis buffer. After resuspension, the mix was centrifuged for 1 hour at 40000 rpm in a 45 Ti rotor and the supernatant collected. The supernatant was loaded onto Flag resin (Sigma, A2220), typically 3 ml dry-bead volume per 100-200 ml of extract, washed with ~30 ml wash buffer 1 (50 mM HEPES pH 7.6, 400 mM ammonium sulfate, 1 mM EDTA, 10 mM MgSO_4_) and then 30 ml wash buffer 2 (50 mM HEPES pH 7.6, 500 mM NaCl, 1 mM EDTA, 1% Triton, 0.1% Na-deoxycholate). Subsequently, 3 ml elution buffer (50 mM HEPES pH 7.6, 110 mM KOAc, 5 mM MgOAc, 1 mM EDTA, 0.1 mg/ml FLAG peptide) was added and incubated with the resuspended beads for 15 min with rotation. The eluate was collected, and the elution repeated 4 more times. The FLAG elutions were combined and loaded onto a MonoQ column (Cytiva, 17516601), followed by gradient elution from buffer A (50 mM HEPES pH 7.6, 110 mM KOAc, 5 mM MgOAc, 1 mM EDTA, 10 mM DTT) to buffer B (same as A with 2 M KOAc). The peak fractions containing pure RNAPII were combined and dialyzed against MonoQ buffer A by Vivapsin 20 concentrators (Sigma, Z614661). Finally, glycerol was added to 5% before aliquoting and snap freezing in liquid nitrogen, prior to storage at -80°C. TFIIS and calf thymus RNAPII were purified as previously described.^85^

### Rat1-Rai1 purification

Plasmids pZH81 and pZH82 expressing Rat1-His and Rai1 were transformed into BL21-CodonPlus (DE3)-RIPL (Thermo Fisher Scientific, NC9122855). Cells were grown at 37 °C to OD_600_ at around 0.6 and induced by the addition of 0.15 mM isopropyl-ß-D-thiogalactopyranoside (IPTG). Cells were further cultured overnight at 20 °C and harvested. Pellets of Rat1-His and Rai1 expressing cells were combined for co-purification. Rat1-Rai1 was purified as previously described.^14^

### Spt4-Spt5 expression and purification

Bacmids were isolated from *E. coli* DH10Bac (Thermo Fisher Scientific, 10361012). Each bacmid was verified by PCR for the genes of interest. P1 and P2 viruses were produced according to the manufacturer’s instructions. P1 and P2 were kept at 4 °C. Large-scale expression cultures were set up by co-infecting virus expressing His-Spt4 and Spt5 in 500 ml Sf9 suspension cultures at 2 million cells per mL with 1% volume/volume P2 virus. Following incubation for 48 hours post-infection (140 rpm, 27°C), cells were harvested by centrifugation (1000 × g, 10 min, 4°C), washed in ice-cold PBS, and snap frozen in liquid nitrogen. Cells were lysed by sonication in lysis buffer (20 mM Tris–HCl, pH 8, 300 mM NaCl, 10 mM ZnCl2, 20 mM imidazole, 4 mM 2-Mercaptoethanol, 5% glycerol, Protease inhibitor Roche cocktail tablet) and the soluble extract was loaded onto His-trap column (Cytiva, 11000458) and washed with lysis buffer. Elution was with lysis buffer containing 250 mM imidazole. Fractions containing Spt4-Spt5 were combined and loaded onto HiTrap Heparin HP column (Cytiva, 17040601). A 15-column volume salt gradient from low (20 mM Tris–HCl pH 8, 10 μM ZnCl_2_,2 mM DTT, 5% glycerol) to high salt buffer (same as low with 1 M NaCl,) was used to elute the protein, and fractions containing Spt4-Spt5 were loaded onto a MonoQ column (Cytiva, 17516601). A 15-column volume salt gradient (same buffer as for Heparin column) was used to elute protein and fractions containing Spt4-Spt5 were combined and dialyzed in storage buffer (20 mM Tris–HCl pH 8, 150 mM NaCl, 10 mM ZnCl_2_, 2 mM DTT, 5% glycerol).

### Purification of long DNA oligonucleotides for use in transcription *in vitro*

3 nmol of oligonucleotide (more than 60 nt long) were resolved by 10% native PAGE and revealed by 10 s exposure under epi-blue light using the Azure imaging system (Azure biosystems). The relevant bands were cut out and soaked in ~ 300 μl TE buffer. The sample was frozen for 10 minutes at -80°C or until solid, and then quickly thawed in a 50°C water bath and incubated for 20 minutes.^86^ This freeze and thaw cycle was repeated once. The oligonucleotides in the gel-free supernatant were then recovered by ethanol precipitation, and the concentration determined by Nanodrop (ThermoFisher Scientific). Oligonucleotides used in this study are shown in Table S1.

### Production of fluorescently labelled RNA marker

Fluorescently labelled RNA marker was produced by incorporating Fluorescein-12-UTP (Jena bioscience, NU-821-FAMX) into RNA marker template (Thermo Fisher Scientific, AM7782) via the MAXIscript T7 transcription kit (Thermo Fisher Scientific, AM1312).

### *In vitro* transcription and termination

The elongation complex was assembled as previously described with minor modification.^87^ Briefly, 5 pmol of pre-annealed RNA:DNA (template strand) hybrid was mixed with an equimolar amount of pure RNAPII, followed by the addition of 10 pmol 5’ biotin-labelled non-template strand DNA, and incubation.^87^ The assembled elongation complex was then immobilized onto streptavidin beads T1 (Thermo Fisher Scientific, 65602) and washed with transcription buffer (TB) containing 20 mM Tris-HCl pH 7.5, 100 mM NaCl, 8 mM MgCl2, 10 μM ZnCl2, 10% glycerol, 2 mM DTT, then with TB/0.1% Triton, TB/0.5 M NaCl and finally TB buffer. NTPs were added into the reaction to initiate elongation at 30 °C. TB with either 0.1 or 0.3 M NaCl was used in the reactions. Briefly, the reactions with Rat1-Rai1 or Spt4-Spt5 were performed at 0.1 M NaCl, while most other transcription and termination tests were performed at 0.3 M NaCl, which increases spontaneous termination efficiency. Reactions were stopped by adding EDTA (to 25 mM final concentration) and separated into supernatant and bead fractions. The bead fraction was resuspended in 8 μl of loading buffer (13 TBE, 8 M urea) and boiled for 5 min at 95° C, while RNAs in the supernatant fractions were first ethanol precipitated before resuspension in 8 μl of loading buffer. The samples were subjected to 6-15% denaturing PAGE (8 M urea) and the results visualized by fluorescent imaging or phosphorimaging using a Typhoon scanner (GE healthcare).

For long transcription templates, TEC assembling using oZH102, oZH103 and oZH104. Then the long transcription template containing dual G-less cassette and terminators was PCR-amplified from related plasmids (pZH20 and pZH23) by using primers containing BbsI restriction digestion sites (oHZ106 and oZH39). The PCR product was digested with BbsI and ligated to the preassembled elongation complex by T4 DNA ligase (NEB, M0202L).

For step walking experiments of RNAPII, beads were washed 5 times with 100 μl TB (100 mM NaCl) between each step to ensure the complete removal of nucleotides. A 2 min incubation was used to for each step of walking. The success of such RNAPII walking depends on the template sequence; some sequences tend to induce pausing/arrest/backtracking of RNAPII and thus hinder RNAPII from moving forward. For Rat1-related assay, transcription termination is determined by the release of RNAPII. In these assays, to prevent RNAPII Run-off, TECs that read through the termination sequence are stalled at specific sites downstream by omitting distinct nucleotides. Rat1-Rai1 cannot degrade single-stranded DNA, so a chimeric DNA-RNA oligo was used to prevent RNA degradation and thus test the torpedo model.

### Western blot

Proteins were separated on 4%–12% NuPAGE (Thermo Fisher Scientific, WG1403A) and transferred to a PVDF membrane (Merckmillipore, IPFL00010), which was blocked in Intercept (PBS) blocking buffer (LI-COR, 927-70001) for 1 h at room temperature. Incubation with primary antibodies in blocking buffer was overnight at 4°C. Membranes were washed several times in PBST, incubated with fluorescent dye-conjugated secondary antibody in blocking buffer for 45 min at room temperature, and washed several times in PBST. Signal detection was by typhoon FLA 9500 using near infrared (NIR) settings.

### Denaturing PAGE Purification of DNA and RNA Oligonucleotides for smFRET

All oligonucleotides for smFRET were purchased from IDT (Integrated DNA Technologies) and purified via denaturing 18% Urea-PAGE before labelling. Oligonucleotides were either with a 5’ modified C6-NH2 group or an internal dT-C6-NH2 for labelling. Modified oligonucleotide (25 μL) was incubated with the NHS-ester Cy3 or Cy5 (GE Healthcare) dissolved in 10 μL of DMSO, 15 μL of NaHCO3/Na2CO3 (9:1) buffer and incubated overnight at 4°C.^88^ Finally, labelled and unlabeled oligonucleotides were separated by reverse-phase HPLC on a C8 column and eluted using triethylamine acetate (TEAA) buffer pH7.0. Labelled product was dried using a vacuum concentrator (Eppendorf) and the final concentration was determined by UV absorbance at 260nm.

### Slide passivation and smFRET

Quartz slides and coverslips were cleaned and passivated as previously described.^89^ Slides were assembled as previously described.^90^ Imaging chambers were first washed with T50 buffer (50 mM Tris-HCl, pH 7.5, 50 mM NaCl) before incubation with neutravidin (0.2 mg/mL) for 10 min. Excess neutravidin was washed off with 1x transcription buffer (TB, 20 mM Tris-HCl pH 7.5, 100 mM NaCl, 8mM MgCl2, 100 μM ZnCl2, 2 mM DTT). Biotinylated DNA/RNA hybrids were assembled by annealing in TB at 90°C for 2 min before slow cooling to RT. Biotinylated DNA/RNA hybrids labelled with Cy3 and Cy5 respectively were immobilized on the surface for 10 min at 10 pM. Non-bound DNA/RNA hybrids were washed out with imaging buffer (1x TB, 2.5 mM PCA, 50 mM PCD, 0.2 mg/mL BSA, Trolox). Next, RNAPII was incubated in the imaging chamber to allow binding to the DNA/RNA hybrid for 30 min followed by incubation of the NTS for another 20 min to assemble the TEC. Pre-transcription FRET was recorded at either 60 or 100 ms framerate using green laser (532 nm, ~2-3 mW) excitation. To measure transcription in real time, NTP’s (10 or 100 μM) were flowed into the imaging chamber and FRET was recorded under the same conditions as before for 5 mins. All measurements were acquired on a home-built prism-based total-internal reflection fluorescence (TIRF) microscope. FRET efficiencies were calculated using a home-built script as a ratio of (acceptor intensity)/(total acceptor and donor intensity)^82^).

### Single-Molecule Trajectories Analysis – ebFRET, TDP and PSH

Single-molecule FRET trajectories were further analyzed using ebFRET^81^ to identify FRET states using >100 trajectories for all conditions tested (as in Figures 3D and 3E, left panels) (https://github.com/ebfret). Hidden Markov modelling identifies discrete states within noisy FRET time trajectories and defines transition probabilities between the observed states. ebFRET employs an empirical Bayesian method to determine the maximum likelihood model based on both a prior and posterior distribution. Model selection can then be carried out to choose the model that yields the best agreement between the data and model. The resulting FRET states can be binned to generate a FRET histogram (0.02 FRET bin size) that recapitulates all of the observed states (as in Figures 3D and 3E, right panels). In addition, FRET trajectories of transcribing molecules were post-synchronized (as in Figures 3D and 3E, middle panels) from the initial 0.9 FRET state from 10 s before the transcription starting point using a MATLAB script (kindly provided by J. Puglisi, Stanford University).^88,91^ Post-synchronization histograms (PSH) were built by binning the smFRET using 0.05 FRET bins, 10 s time bins and a 0.75 FRET threshold to exclude false decreases due to noisy signals. These states appear “blurred out” in the post-synchronization histogram because each state transition occurs at a different stochastic time. All Matlab and IDL scripts used for data extraction and analysis can be found on the Rueda’s github page (https://github.com/singlemoleculegroup). All extracted FRET time traces used to generate the single-molecule data within this paper can be found on the OSF server (osf.io/8zwu9).

### RNA 3’ end sequencing

3’end sequencing experiments were performed as previously described with minor modifications.^44,92–94^ 4-thiouracil (4TU) labelling was performed as previously described.^95^ Briefly, 50ml *S. cerevisiae* cells were grown in YPD medium to OD_600_ around 0.5. Rapamycin (final concentration at 0.1 μg/ml) was added to anchor away Rat1 or Ysh1 for one hour. Then, 4TU (2 M stock in DMSO) was added to the media at a final concentration of 5 mM, and cells were harvested after 6 min of labelling. RNAs were prepared with hot phenol extraction method.^96^ Briefly, cell pellets were suspended in TES buffer (1% SDS, 5 mM EDTA, 10 mM Tris pH 7.5) and extracted twice with phenol (pH 4.3) at 65 °C with shaking for 30 min and once in chloroform at room temperature for 5 min. RNAs were precipitated with ethanol in 20-30 mM LiCl and resuspended in deionized water. Isolation of newly synthesized RNAs using 4TU labelling was performed as in^44,93^ with minor modifications. Briefly, 400 μg RNA was incubated with MTESA biotin-XX linker (Biotium, BT90066) in the biotinylation buffer (10mM Tris pH7.4-7.5, 1mM EDTA, 400ug RNA, 40ug MTSEA biotin-XX linker) in the dark at 24 °C for 30min with 750 rpm shaking. After biotinylation, Chloroform/Isoamyl alcohol (24:1) was applied to remove excess MTSEA biotin-XX liner. After purification, 200 μL of μMACS streptavidin MicroBeads (Miltenyi Biotec, 130-074-101) was added to bind biotin labelled RNAs and isolate them. The RNAs eluted from streptavidin beads were further cleaned up by RNeasy minElute kit (Qiagen, 74204). To efficiently capture <200 nt fragments from the MinElute spin columns, the amount of ethanol added to the RNA and RLT buffer was increased compared to the manufacturer’s recommendation. For a 200 μL sample, 700 μL of RLT buffer and 1,050 μL of 100% ethanol was added, mixed well and applied to the minElute spin columns over three rounds. The remaining protocol was as recommended by Qiagen. The RNA was eluted in 20 μL. Where relevant, *in vitro* polyadenylation was performed by using *E. coli* polyA polymerase (EPAP) (NEB, M0276L). Ribosome RNAs were depleted by using RiboMinus Transcriptome Isolation Kit (Thermo Fisher Scientific, 10388792). Library of 3’ end sequencing was prepared by using Lexogen QuantSeq 3’ mRNA-Seq Library Prep Kit REV (Lexogen, 16.24) for Illumina and sequenced with single end 75bp reads by NextSeq 500/550 Mid Output Kit v2.5 (150 Cycles) with advanced sequencing science technology platform (STP) at the Francis Crick institute.

### Quantification And Statistical Analysis

As indicated in the figure legends, data values reported in the figures are the mean ± standard deviation using Microsoft Excel. The statistical and analytical details of the experiments are provided in the bioinformatic method details below.

### Poly-A/C/T/G tract sequence analyses of the *Saccharomyces cerevisiae* genome

A set of open reading frames (ORFs) was defined based on the Ensemble release 104 transcript annotation of the *Saccharomyces cerevisiae* R64-1-1 genome assembly. Where multiple ORFs existed per gene, only the single largest was selected (n=6600). The ends of the ORFs were defined as the end of the codon prior to the STOP codon. The genomic intervals representing each ORF were extended 500bp downstream in a strand-aware manner. The extended ORF regions were then scanned for runs >=5bp of the same base (A,C,T,G), recording their maximal lengths and genomic positions. Matches that could not be resolved to a single gene were discarded, i.e., those that mapped to more than one extended ORF on the same strand, or those that mapped to convergent overlapping ORFs from opposing strands. Matches to ORFs from mitochondrial genes were also discarded. The frequency of homo-bp runs between 5-20bp overlapping i) the original ORF definitions or ii) the 500bp downstream flanking region was subsequently calculated for each base.

### Poly-A/C/T/G tract sequence analyses of termination sites

The positions of 903 yeast termination sites defined by Baejen et al.^41^ were downloaded from the NCBI’s Gene Expression Omnibus (GSM2199309). A set of genomic intervals representing the +/-500bp region flanking the termination sites was defined and the sequence scanned for the largest run of consecutive homo-nucleotides (A, C, T or G) in a strand-specific manner. The identity and position of just the 3’ end of each nucleotide run was recorded and used to construct a counts matrix across all 903 search regions. Each search region was subsequently divided into 100 equally sized bins and a sum of all counts for each base per bin was plotted.

### 3’ end sequencing alignment, quantification and normalization

Reads were trimmed of their Illumina TruSeq single index adapter sequences using Cutadapt v2.10,^75^ allowing for reads with a minimal length of 20bp to remain (–minimum-length 20 -a AGATCGGAAGAGCACACGTCTGAACTCCAGTCA). Trimmed reads were aligned against the *Saccharomyces cerevisiae* R64-1-1 genome assembly using STAR v2.7.6a^76^ with default settings. Low quality, multi-mapping alignments were removed from the downstream analysis using Samtools v1.122 (-q 255). Similarity between biological replicate alignments was assessed using deepTools v3.3.1’s2 multiBamSummary function. Coverage between samples was compared across consecutive 1kb genomic bins using a Spearman correlation coefficient. Reads from individual biological replicates were merged into single files for the purposes of visualization. Per-million scaled, strand-specific BedGraph files representing read-depth coverage of the most 3’ transcribed base of reads from each sample were created from the filtered BAM files using BEDTools v2.27.1’s2 genomecov function (-bg -5 –strand [+-] –scale [scale factor]). Note that due to the library preparation being “reverse”, it was necessary to request the 5′ base from genomecov. Scale factors were calculated as 1,000,000 divided by the total number of mapped reads. BedGraph files were subsequently converted to Bigwig format using Kent Tools2 bedGraphToBigWig function.

### Related annotation files

Putative polyA sites from 3′-end sequencing of yeast poly(A)+ RNA^92^ were downloaded from GEO as strand-specific BedGraph files (GSM1959710). Sites were filtered to select just those with an associated score >3 and further reduced into a set of non-overlapping genomic intervals. Where possible, each interval (i.e., polyA site) was assigned to an upstream gene based on a proximity to the 3’ end of the CDS of <=500bp and consequently given that gene’s strand orientation. After filtering 4902 genes were assigned a polyA site. The 1kb region downstream of the assigned polyA sites was scanned for nucleotide tracts of a least 5, 6, 7 or 8 consecutive resides of the same type (i.e., A, C, T or G). Tracts identified within 50bp of a putative polyA site (score>3) or any that overlapped with a snRNA, snoRNA, rRNA or tRNAs were discarded. Tracts passing the filtering step were profiled for bp-level sense read-depth coverage in the scaled bigwig files across the region +/-20bp of their 3’ ends using the computeMatrix function from deepTools v.2.5.3. A subset of cryptic unstable transcripts (CUTs) was selected according to transcription level2. Independently transcribed monocistronic sn/snoRNAs were selected for the sn/snoRNA subset.

### Heatmaps and metagene profiles

Heatmaps and lineplots depicting coverage were created using deepTools’ plotHeatmap function. In situations where the profiles looked noisy, an additional run of computeMatrix was performed using a set of blacklisted regions representing outlier tracts. The blacklisted regions were created by identifying the tracts containing the top 100 most extreme positive values from the original coverage matrix in each sample. A combined set of outlier tracts from all samples was aggregated as a single backlist which was used as an additional argument to computeMatrix and thus ensure that the same tracts were consistently profiled across conditions. Similar plotHeatmap methods were also applied on Europe Galaxy server (https://usegalaxy.eu) to produce heatmaps aligning to 3’end of CUTs, sn/snoRNAs and introns.

### Motif analysis of spontaneous termination sites

A set of candidate spontaneous termination sites was defined by identifying regions 1 kb downstream of protein-coding genes that showed enriched coverage in Ysh1AA relative to WT conditions. More specifically, a new set of strand-specific bigwig files representing a ratio of the normalized, strand-specific, 3’ bigwig files of the Ysh1AA and WT conditions was created using deepTools’ bigwig-Compare function (–operation ratio, –pseudocount 3, –binSize 1). A pseudo-count of 3 was added to each value in each file prior to ratio calculation to avoid infinite values. Termination sites were then defined as the intervals within the 1kb regions downstream of protein-coding genes that showed a ratio >2 between Ysh1AA and WT conditions. Termination sites were assigned a strand based on the upstream gene. Then the 10nt of sequence upstream of termination sites was subjected to motif discovery and enrichment analysis for motifs 5-10bp in width using the findMotifs.pl function from the HOMER^51^ suite v4.10.42 against a background of the 10nt downstream of the termination sites. (findMotifs.pl upstream.fa fasta output_dir -fasta downstream.fa -norevopp -nomask-nlen 0 -noweight -len 5,6,7,8,9,10.)

## Supplementary Material

Supplemental information

## Data Availability

Genome-wide data that support the findings of this study have been deposited in GEO (https://www.ncbi.nlm.nih.gov/geo/) with the accession code GSE218928. The original images of the study are at Mendeley, [https://doi.org/10.17632/xwfh7rnzc2.1] any additional information required to reanalyze the data reported in this paper is available from the lead contact upon request.

## In brief

Han et al. use reconstitution of transcription and single-molecule analysis to show that RNAPII pauses and then spontaneously terminates at terminator sequences containing T-tracts. The “torpedo” Rat1-Rai1 exonuclease greatly stimulates spontaneous termination, whereas elongation factor Spt4-Spt5 suppresses it. Termination at T-tracts in cells is confirmed by genome-wide analysis.

## Highlights

- RNAPII terminates spontaneously at terminator T-tracts *in vitro*
- Spontaneous termination involves prolonged pausing without backtracking
- Torpedo exonuclease Rat1 cooperates with T-tracts to elicit efficient termination
- 3′ end sequencing confirms T-tract directed termination *in vivo*
