# Supplementary material for: DNA-directed termination of RNA polymerase II transcription
Source: Mol Cell. Author manuscript; Available in PMC 2024 Feb 15. (PMC7615648; doi:10.1016/j.molcel.2023.08.007)
Supplement: Supplemental information [file EMS193944-supplement-Supplemental_information.pdf]

Supplementary Figures with legends

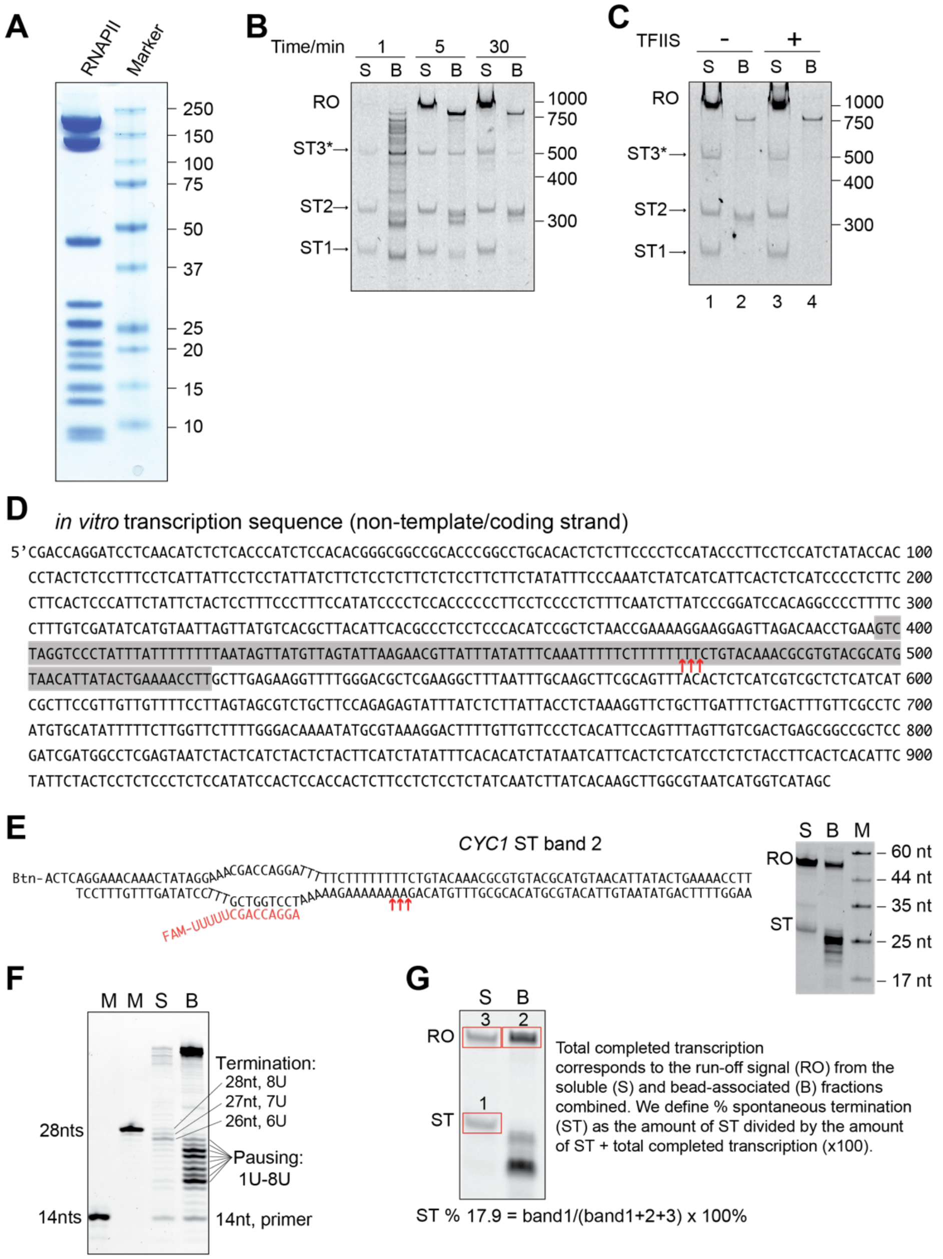

Figure S1. Detection of eukaryotic RNAPII termination, related to Figure 1. A. SDS-PAGE and Instant Blue staining of RNAPII purified from *S. cerevisiae*. **B.** Analysis of *in vitro* transcription and termination using 5'-end Fam-labelled RNA, by 6% TBE-Urea polyacrylamide gels. RO, run-off; S, supernatant; B, beads; ST, Spontaneous termination; ST3*, is due to plasmid backbone sequence. **C.** Effect of elongation factor TFIIS on spontaneous termination, analyzed as (B). **D.** Sequence of *CYC1* terminator. Red arrows indicate the spontaneous termination sites. Grey region indicates the approximate termination sequence. **E.** Left, Sequences used for TEC assembly; Red arrows indicate the spontaneous termination sites; Right, 5'-end FAM-labelled RNA transcripts were resolved and visualized as in (B). **F.** 5'-end FAM-labelled RNA transcripts were resolved in a longer gel; 14 nt and 18 nt RNA markers with the template sequence show precise transcript lengths. **G.** illustration of how spontaneous termination efficiency (%) is calculated.

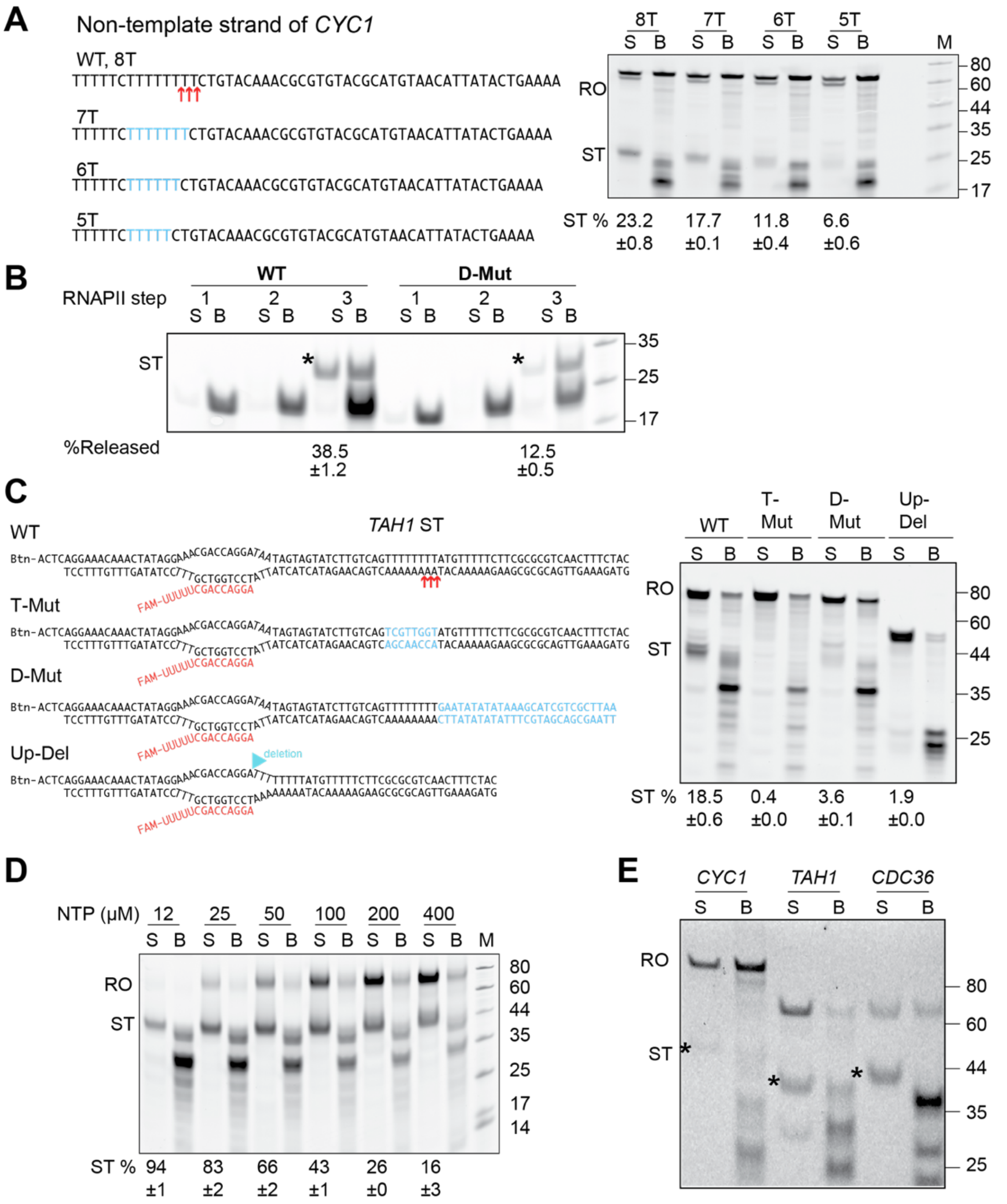

Figure S2. Characterization of RNAPII termination, related to Figure 2. A. Right, Non-template (coding strand) sequences. TECs assembled as previously. Altered sequence in light blue. Red arrows indicate spontaneous termination sites. Right, analysis as in Figure S1B. **B.** Similar to Figure 1C; step walking of RNAPII on WT *CYC1* ST (left) or D-Mut sequence (right, see (C)), comparing the stability of the TEC when paused at the T-tract. % spontaneous termination shown, n=3. **C. Left,** Sequences used for TEC assembly from the termination region of the *TAH1* gene. Red arrows indicate the sites of termination. Altered sequence in light blue. T-Mut, T-tract was mutated to GC; D-Mut, mutation of Downstream sequence of T-tract; Up-Del, Deletion of Upstream sequence of T-tract. **Right,** analysis as in (A). **D.** Effect of NTP concentration on spontaneous termination, executed as in S2A using WT, 8T template. **E.** *In vitro* transcription and termination by calf thymus RNAPII (CT RNAPII). Analysis as previously. Asterisk indicates spontaneous termination bands.

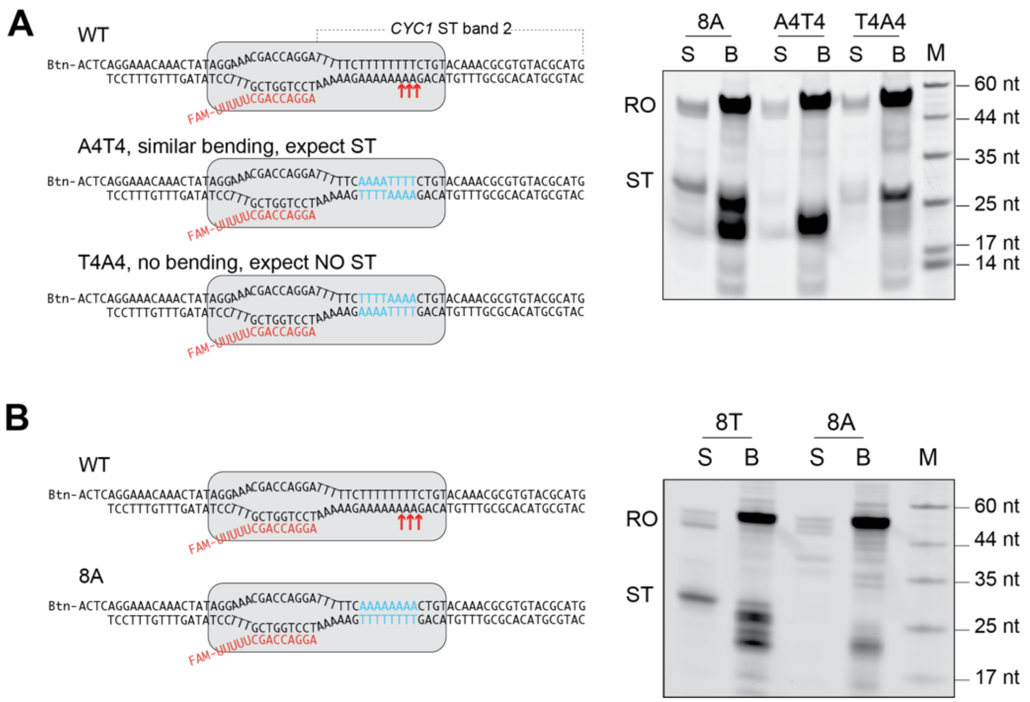

Figure S3. DNA bending is not the cause for spontaneous termination, related to Figure 2. A. Left, sequences for TEC assembly, bending or non-bending. Altered sequences in blue. Red arrows indicate sites of spontaneous termination. **Right,** Analysis as previously. **B.** As in (A), but inversion of A/T tract.

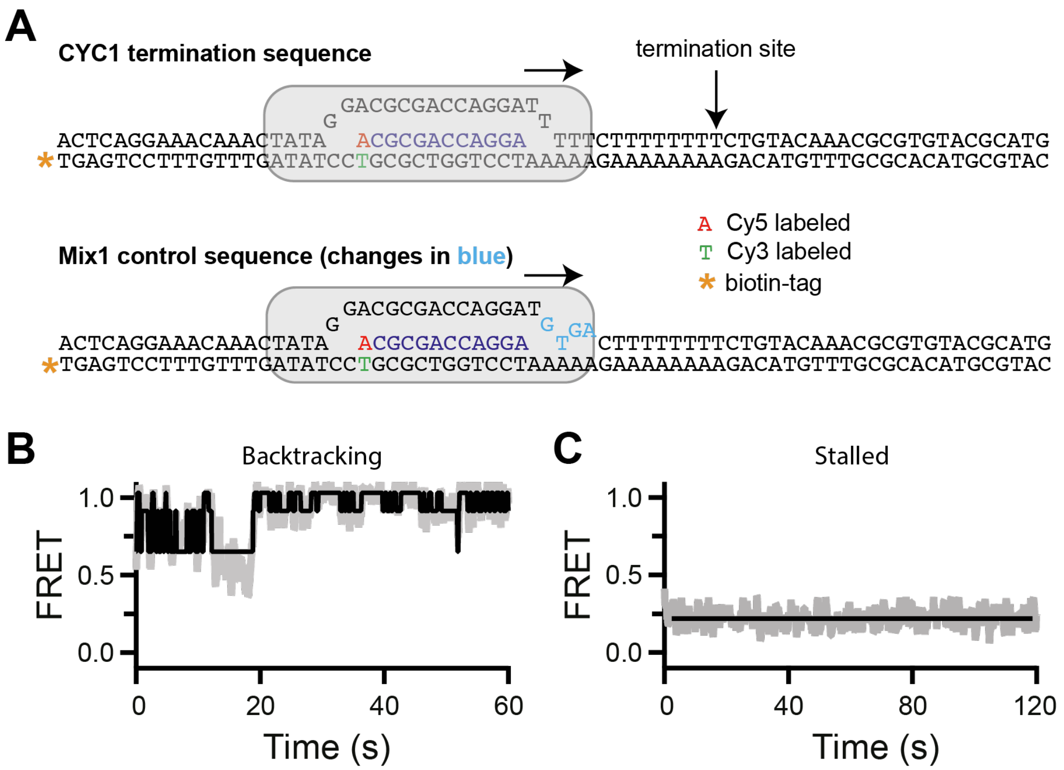

Figure S4. smFRET analysis of termination, related to Figure 3. A. Schematic of smFRET assay with nucleic acid sequences. **B.** FRET trace example of backtracking. ebFRET fitted data shown in black. **C.** As in (B), but an example of stalled RNAPII. Examples of polymerases finishing transcription (final FRET=0) are shown in Figure 3D and E.

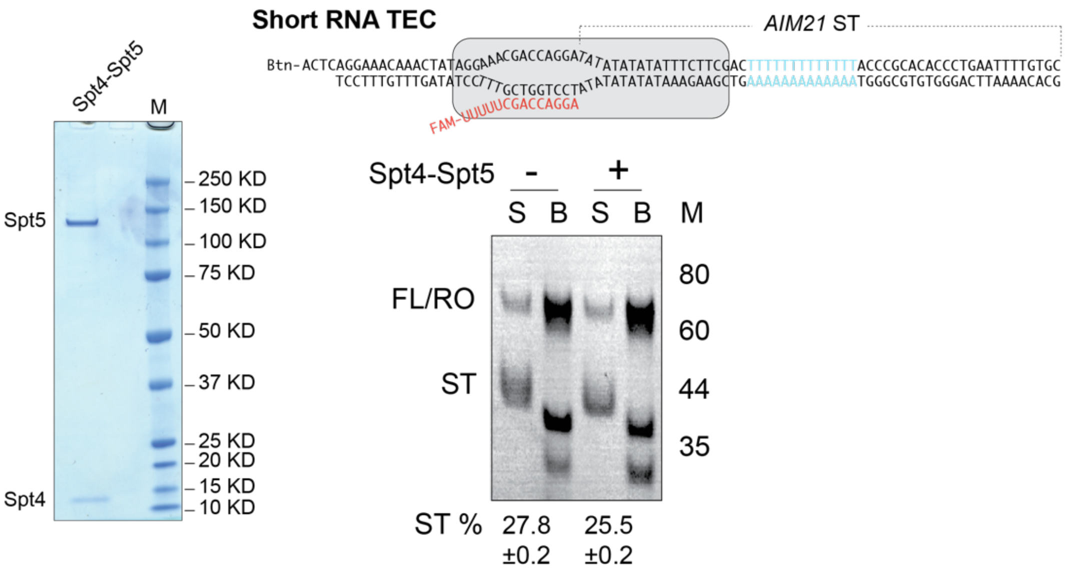

Figure S5. Spt4-Spt5 complex does not work on a short transcript, related to Figure 4. Left, SDS PAGE and Instant Blue staining of Spt4-Spt5 complex purified from Baculovirus-infected *Sf9* insect cells. **Right,** performed as in Figure 4.

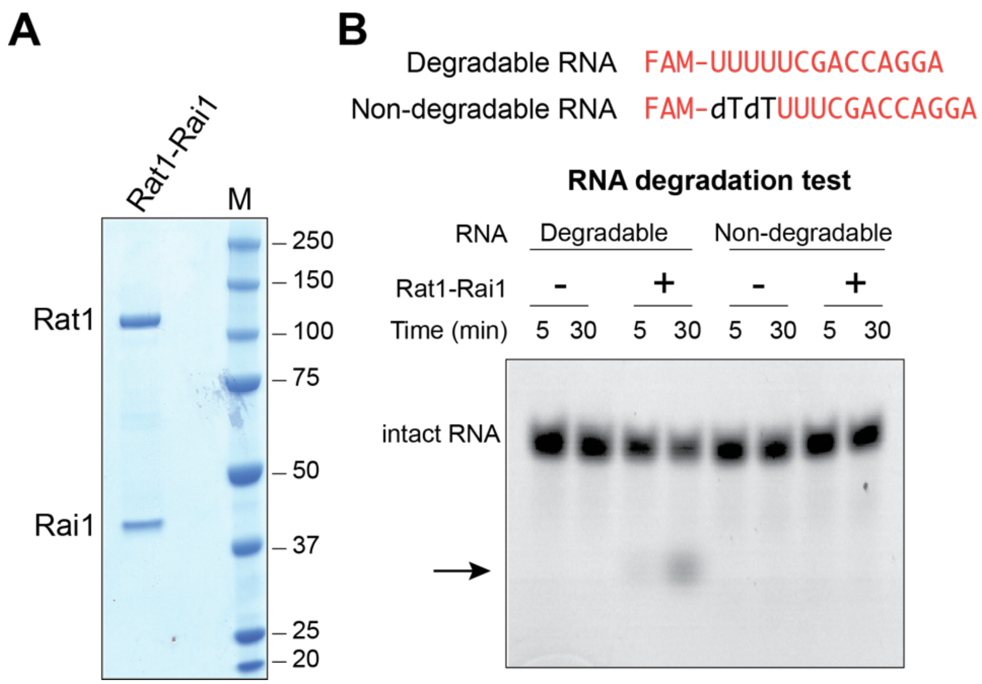

Figure S6. Rat1-Rai1 RNA degradation test, related to Figure 5. A. SDS-PAGE and Instant Blue staining of Rat1-Rai1 co-purified after expression in *E. coli*. **B. Upper,** Sequence of degradable RNA with 5' Fam labelling and non-degradable chimeric RNA with the first two Us mutated to dT; **Lower,** RNA degradation test. Mixing RNA with Rat1Rai1 to check whether the RNA was degradable. After incubation, Fam-labelled RNAs were visualized by 15% TBE-Urea PAGE. Note that 5' Fam labelled RNA is degraded by Rat1-Rai1 only on the degradable substrate.

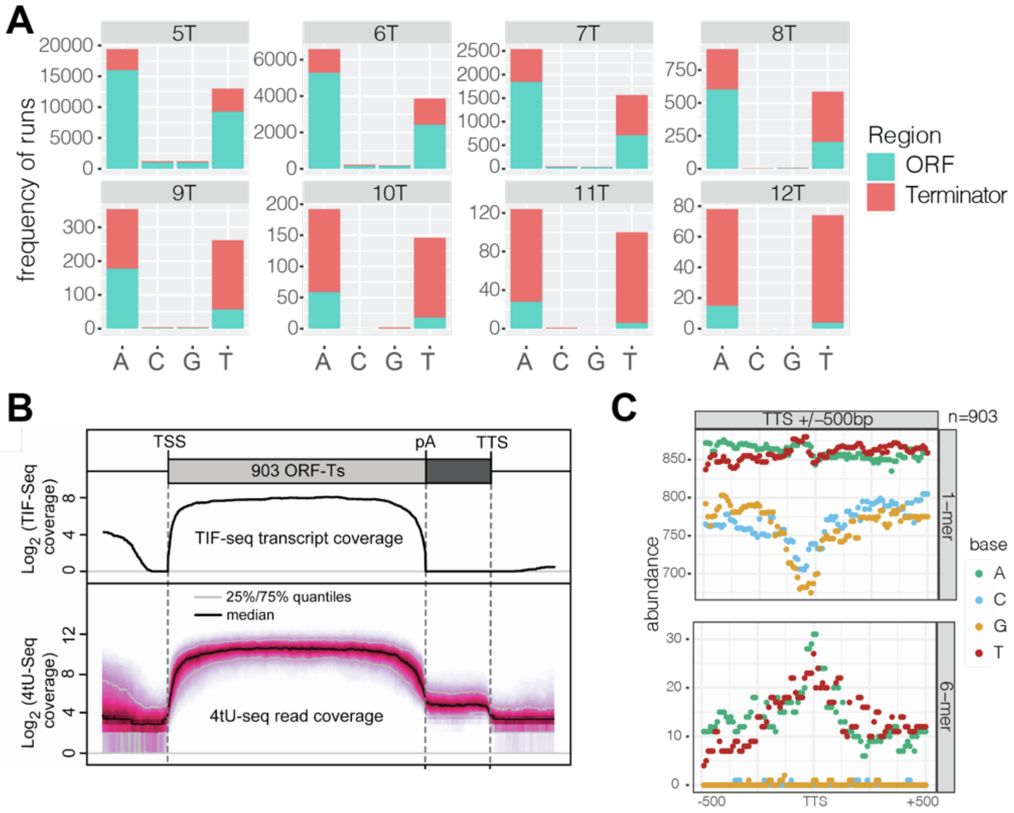

Figure S7. Long T-tracts are mainly located in RNAPII gene terminators, related to Figure 6. A. Sequence analysis of yeast *Saccharomyces cerevisiae* genome and charting of poly-A/C/G/T tracts in ORFs and in the termination region. **B.** Figure from Baejen et al., 2017[1], in which RNAPII transcription termination sites of in subset of 903 yeast genes are indicated by a sharp decrease of 4TU-seq signal downstream of polyA sites. **C.** Sequence analysis of the 903 sites from B. 1-mer means single nucleotide sequence analysis around TTS, 6-mer means poly-A/C/G/T tracts (≥ 6 mer) around TTS.

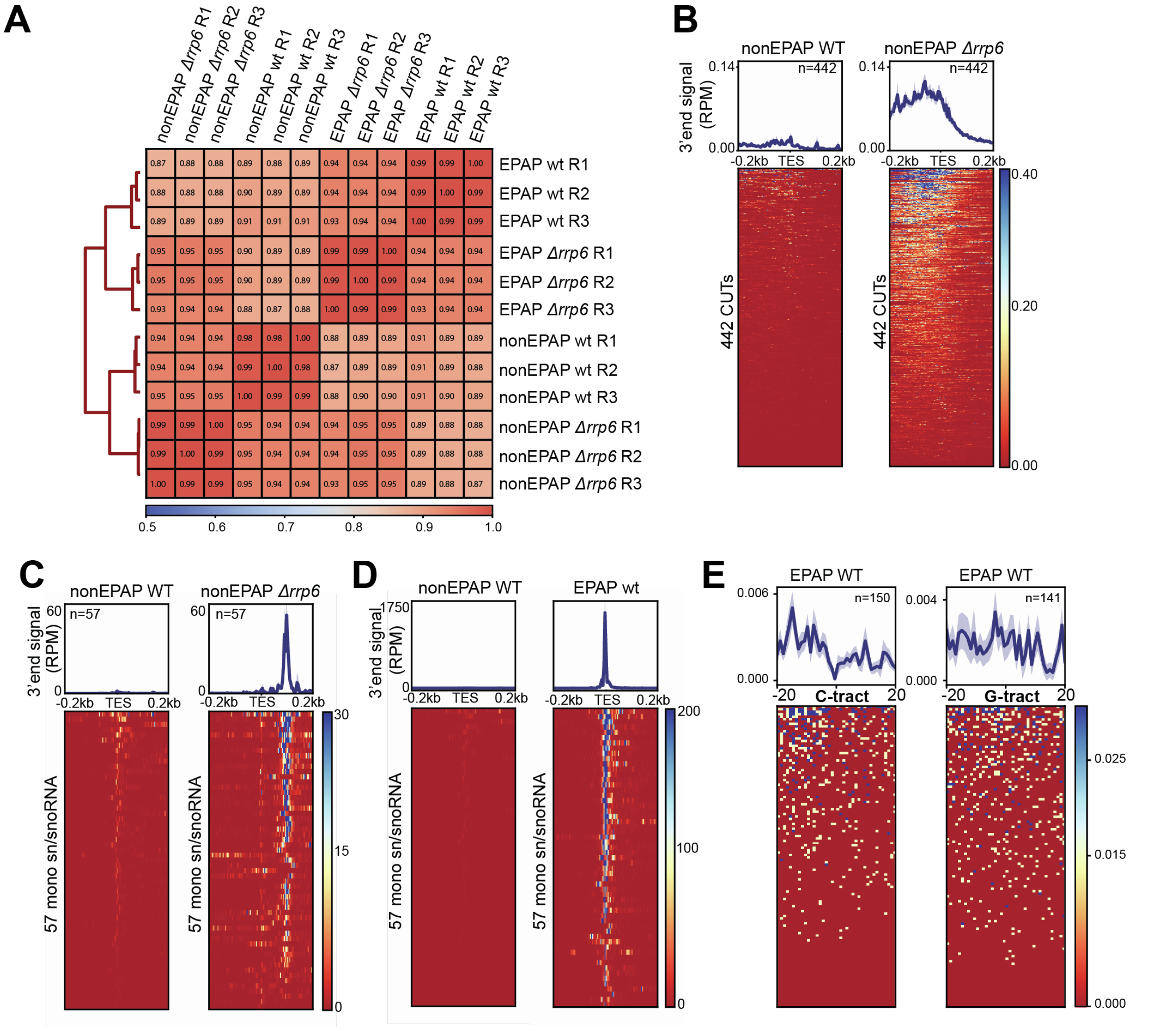

Figure S8. 3'-end sequencing results in wild type and *rrp6* mutant cells, related to Figure 6. A. Reproducibility matrix. **B.** Experiment showing rapid degradation of cryptic unstable transcripts (CUTs) by the Rrp6-exosome, demonstrating that Rrp6 function is indeed disabled in the *rrp6* strain used. Heatmap and metagene profile of 3'-end sequencing signals are aligned to the transcription ending site (TES) of CUTs. The transcription termination region of CUTs is a wide region and CUTs are rapidly degraded by Rrp6-exosome. **C.** Rapid degradation of sn/snoRNA precursors by Rrp6-exosome. Heatmap and metagene profile of 3'-end sequencing signals aligned to the TES of mature sn/snoRNAs for WT and *Δrrp6* without *in vitro* polyadenylation. Transcription termination of sn/snoRNA happens at downstream of the mature sn/snoRNA, and the precursors are quickly processed by Rrp6-exosome to form the mature sn/snoRNA. **D.** Experiment showing that EPAP polyA-tailing *in vitro* worked. Heatmap and metagene profile of 3'-end sequencing signals aligned to the TES of mature sn/snoRNAs. Mature sn/snoRNAs do not normally contain polyA tails. **E.** Alignment to C- and G-tracts.

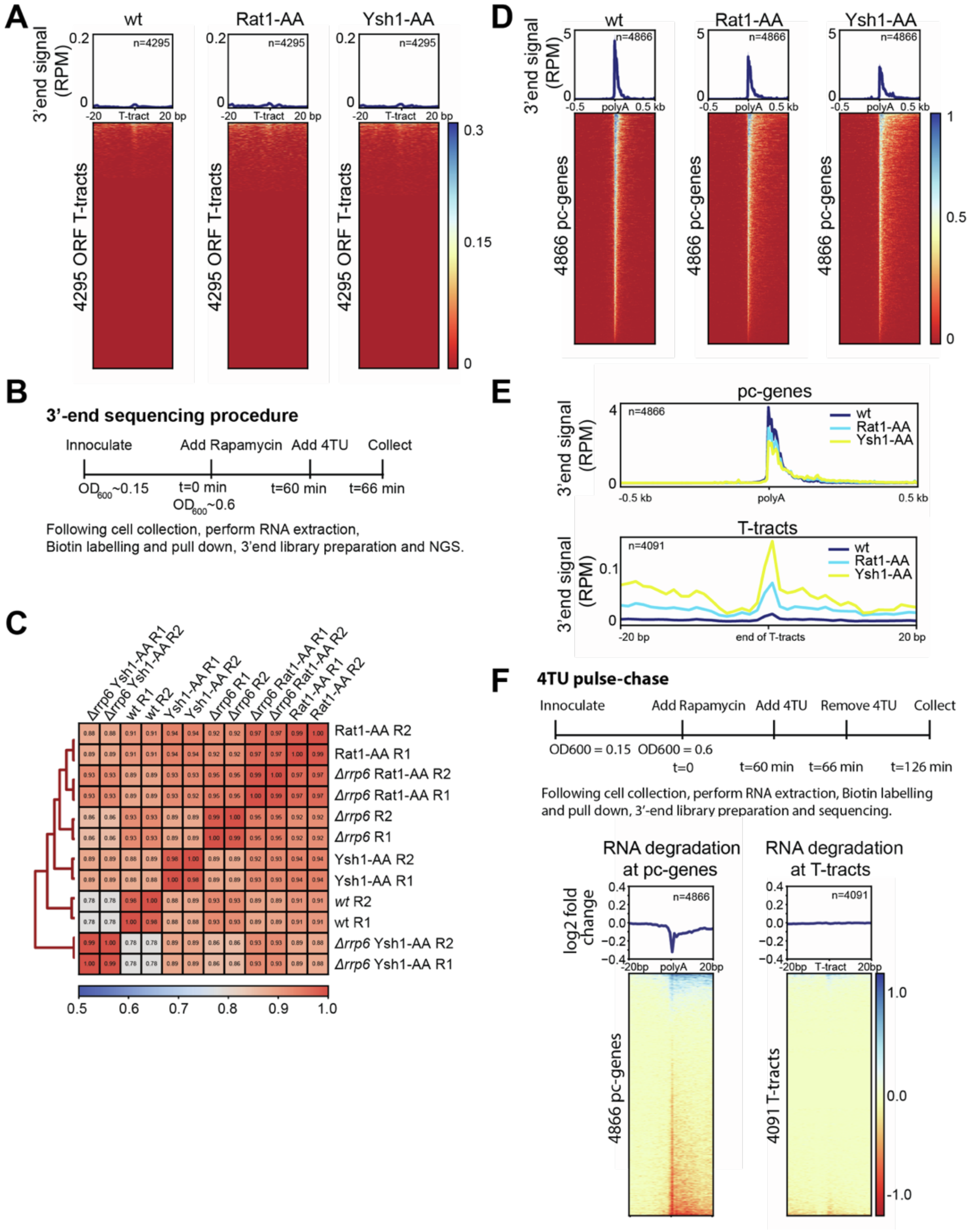

Figure S9. 3'-end sequencing results in anchor-away mutants, related to Figure 6. A. Heatmap and metagene profile of 3'-end sequencing signals aligned to T-tracts (≥ 6 mer) located inside open reading frames (ORFs). Y-scale as in Figure 6C. **B.** Experimental Scheme for RNA 3'-end sequencing experiments. **C.** Reproducibility matrix. **D.** Heatmap and metagene profile of 3'-end sequencing signals, aligned to the polyA site of protein coding genes (pc-genes). **E. Upper**, metagene profiles around the polyA site of pc-genes; **Lower**, metagene profiles for terminator T-tracts. **F.** Pulse-chase experiment showing transcripts released at T-tracts were relatively stable. **Upper**, Experimental scheme. **Lower**, Log2 fold-change of pulse-chase vs no chase was calculated, and heatmap and metagene profiles were aligned to either polyA sites of protein coding (pc) genes, or T-tracts, as indicated. The experiment was performed with the Ysh1-AA strain. Note that for most pc-genes, transcripts amount decreased markedly after one hour chase. The pc-gene transcripts that increased after one hour chase mainly came from very active housekeeping genes, likely because they continued to use the traces of 4TU remaining in cells during the chase, and because these transcripts are very stable.

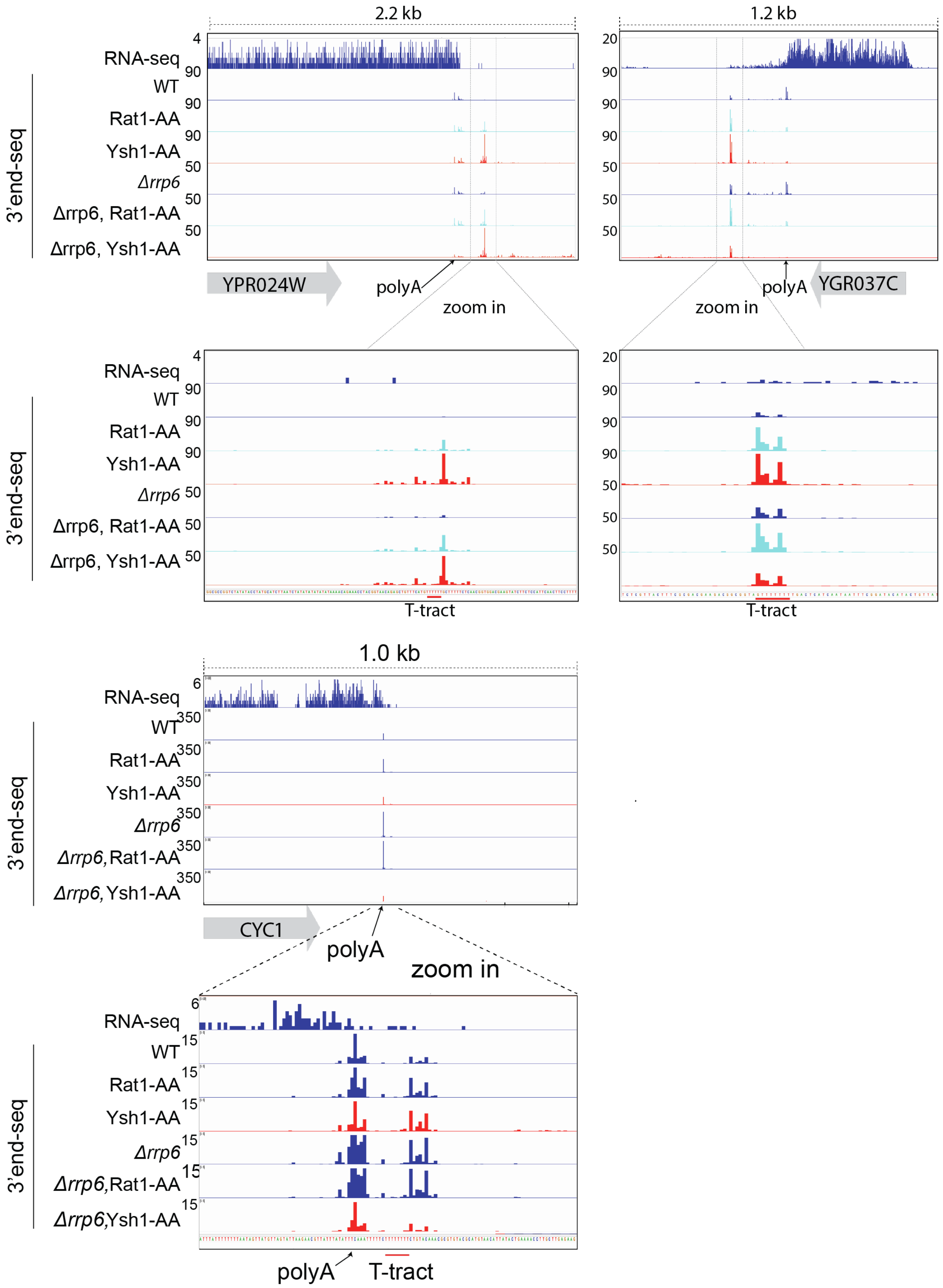

Figure S10. Single gene examples of 3'-end sequencing results, related to Figure 6.

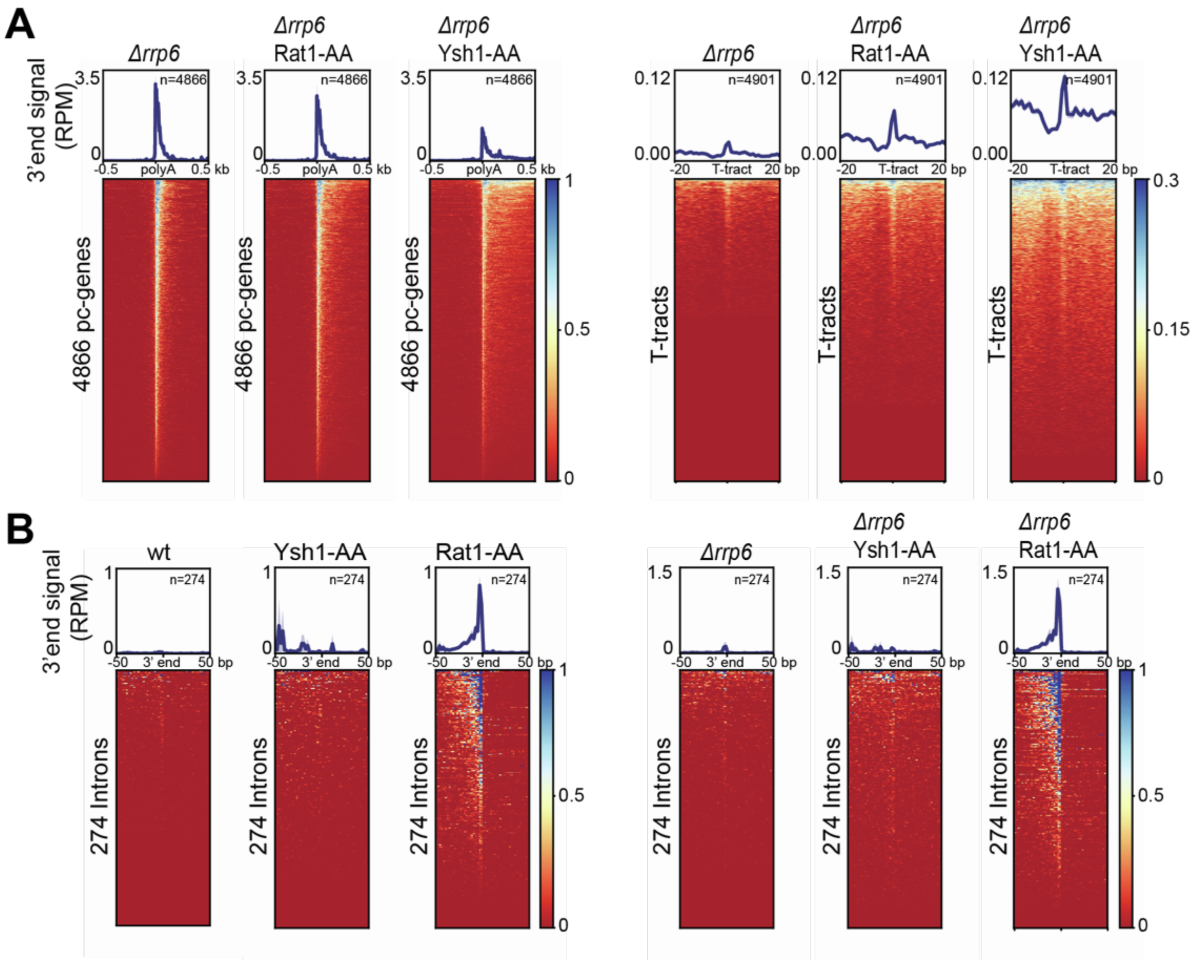

Figure S11. Analysis of 3'-end sequencing results, related to Figure 6. A. Heatmap and metagene profile of 3'-end sequencing signals from *Δrrp6* background were aligned to polyA sites of pc-genes or terminator T-tracts. **B.** Heatmap and metagene profile of 3'-end sequencing signals aligned to 3'-end of introns. Note the stabilization of introns when Rat1 is depleted from the nucleus.
